# Quiescence enables unrestricted cell fate in naive embryonic stem cells

**DOI:** 10.1038/s41467-024-46121-1

**Published:** 2024-02-26

**Authors:** Le Tran Phuc Khoa, Wentao Yang, Mengrou Shan, Li Zhang, Fengbiao Mao, Bo Zhou, Qiang Li, Rebecca Malcore, Clair Harris, Lili Zhao, Rajesh C. Rao, Shigeki Iwase, Sundeep Kalantry, Stephanie L. Bielas, Costas A. Lyssiotis, Yali Dou

**Affiliations:** 1 Department of Medicine, Norris Comprehensive Cancer Center, University of Southern California, Los Angeles, CA 90033 USA; 2 Department of Molecular and Integrative Physiology, University of Michigan Medical School, Ann Arbor, MI 48109 USA; 3 Institute of Medical Innovation and Research, Peking University Third Hospital, Beijing, China; 4 Department of Human Genetics, University of Michigan Medical School, Ann Arbor, MI 48109 USA; 5 Department of Ophthalmology & Visual Sciences, W.K. Kellogg Eye Center, University of Michigan, 1000 Wall St., Ann Arbor, MI 48105 USA; 6 Beaumont Hospital, Wayne, 33155 Annapolis St., Wayne, MI 48184 USA; 7 Present Address: Department of Molecular and Integrative Physiology, University of Michigan Medical School, Ann Arbor, MI 48109 USA

**Keywords:** Pluripotency, Embryonic stem cells, Quiescence

## Abstract

Quiescence in stem cells is traditionally considered as a state of inactive dormancy or with poised potential. Naive mouse embryonic stem cells (ESCs) can enter quiescence spontaneously or upon inhibition of MYC or fatty acid oxidation, mimicking embryonic diapause in vivo. The molecular underpinning and developmental potential of quiescent ESCs (qESCs) are relatively unexplored. Here we show that qESCs possess an expanded or unrestricted cell fate, capable of generating both embryonic and extraembryonic cell types (e.g., trophoblast stem cells). These cells have a divergent metabolic landscape comparing to the cycling ESCs, with a notable decrease of the one-carbon metabolite *S*-adenosylmethionine. The metabolic changes are accompanied by a global reduction of H3K27me3, an increase of chromatin accessibility, as well as the de-repression of endogenous retrovirus *MERVL* and trophoblast master regulators. Depletion of methionine adenosyltransferase *Mat2a* or deletion of *Eed* in the polycomb repressive complex 2 results in removal of the developmental constraints towards the extraembryonic lineages. Our findings suggest that quiescent ESCs are not dormant but rather undergo an active transition towards an unrestricted cell fate.

## Abstract (web summary)

Stem cell quiescence is generally considered as an inactive state with poised potential. Here, Khoa et al. find that quiescent embryonic stem cells actively maintain a dynamic reservoir of cells with unrestricted cell fate that converges on *S*-adenosylmethionine and H3K27me3 status.

## Introduction

Cellular quiescence refers to a state of reversible cell cycle arrest at G_0_ phase, distinct from irreversible G_0_ arrest observed in terminally differentiated and senescent cells^1^. Quiescence is generally considered as a protective mechanism to evade unfavorable external conditions or serve as a reservoir to prevent stem cell exhaustion and aging^2,3^. Many adult stem cells, including hematopoietic stem cells, hair follicle stem cells, muscle stem cells, and neural stem cells, reside in the quiescent state in vivo^1^. They play essential roles in tissue homeostasis and regeneration. The quiescent stem cells are mostly considered “dormant”, capable of rapid exit upon receiving activation or differentiation signals^1,2^. Recent studies including ours have shown that naive mouse embryonic stem cells (ESCs) cultured with leukemia inhibitory factor (LIF) and GSK3β/MEK inhibitors (2i)^4^ can enter a quiescent state in vitro either spontaneously or in response to depletion or inhibition of the histone acetyltransferase MOF (also called KAT8 or MYST1), MYC, or fatty acid oxidation (FAO) pathway^5,6^. In contrast, inhibiting the mechanistic target of rapamycin (mTOR) pathway in both naive and serum/LIF ESCs leads to a diapause-like growth arrest without inducing quiescent G_0_ phase^7^. The quiescent naive ESCs (qESCs) mimic embryonic diapause in vivo, retaining self-renewal ability and developmental potential for three embryonic germ layers^5,6^. Interestingly, cancer cells can also enter an embryonic diapause-like quiescent state to evade cell death from drug treatments^8,9^. These drug-induced cancer persister cells contribute to most cases of relapse in patients and possess a transcriptional signature similar to that of diapausing blastocysts or quiescent ESCs^8,9^. Despite these studies, functional significance of ESC quiescence and how it can be leveraged in regenerative medicine remain unclear.

Preimplantation embryo development is concomitant with remarkable changes in mitochondria. Nuclear distribution of mitochondrial tricarboxylic acid cycle (TCA) enzymes in 2-cell (2C) embryo coincides with global re-setting of chromatin structure and zygotic genome activation^10^. Furthermore, metabolic switch between oxidative phosphorylation and glycolysis coincides with the epiblast development and lineage specification^11^. Naive ESCs derived from the blastocyst are metabolically heterogenous^5,12^. We have shown that naive ESCs with inherently low mitochondrial membrane potential (MMP or ΔΨ_m_) are quiescent^5^. These spontaneous qESCs reside in a reversible G_0_ phase with low metabolic and biosynthetic activity^2,5^. Notably, reduced MMP and adenosine triphosphate (ATP) production are common features of the induced qESCs and adult stem cells as well^5,13,14^. However, whether they have any impacts on the chromatin milieu and the transcriptional landscape in ESCs is largely unknown.

Here we show that the qESCs are functionally distinct from cycling ESCs. The qESCs are able to differentiate into the extraembryonic lineages with high efficiency in vitro. These cells have molecular characteristics akin to 2C embryos, with increased expression of *MERVL*, 2C-specific genes and trophoblast master regulators. We further shed light on the active and coordinated changes of the mitochondrial activity, the one-carbon metabolite *S*-adenosylmethionine (SAM) homeostasis and polycomb repressive complex 2 (PRC2)-mediated H3K27me3 in qESCs during the transition from a restricted to an unrestricted cell fate. Our study suggests that quiescent stem cells are not simply “dormant” or “idle” with poised potential^2^. Instead, they actively undergo the metabolic and epigenetic changes in acquisition of the expanded cell fate plasticity.

## Results

### The spontaneous qESCs have a unique transcriptome similar to that of 2C embryo

**Fig. 1:**
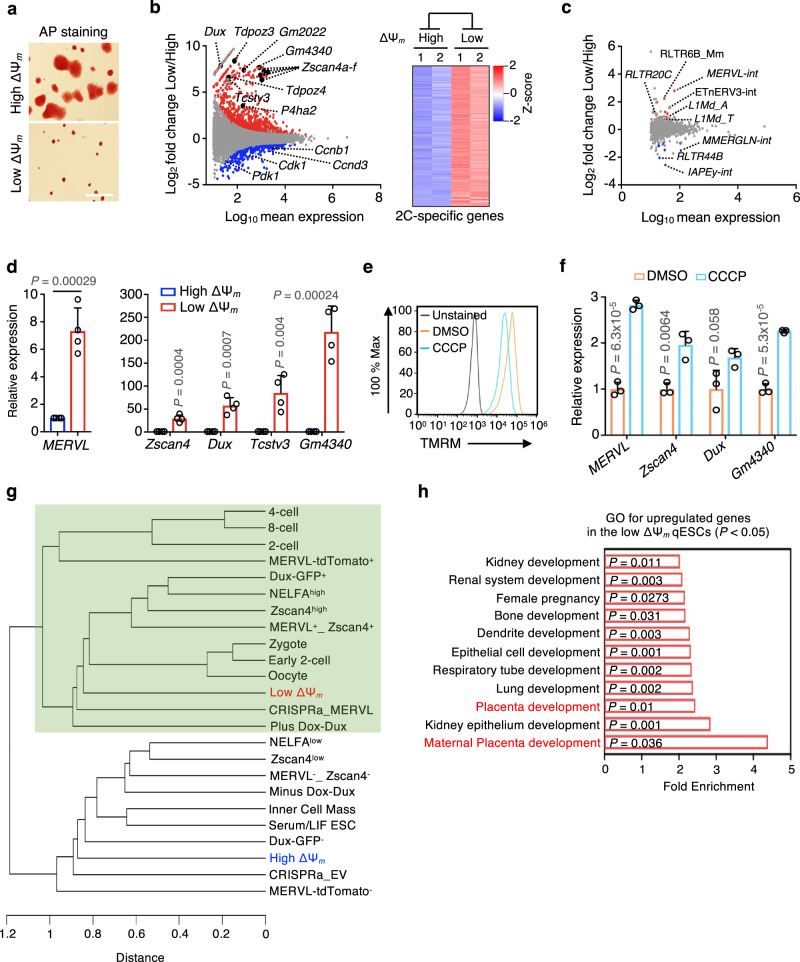
The spontaneous qESCs possess a unique transcriptional landscape. **a** Representative images of AP staining for naive ESCs with top (high) and bottom (low) 5% of TMRM signals at day 4 post FACS sorting. Scale bar, 200 µm. AP Alkaline phosphatase. Images were representatives from three independent experiments. **b** Left, MA plots showing up- (red) and down-regulated (blue) genes in low vs. high ΔΨ_m_ ESCs. Selected 2C and cell cycle genes were highlighted. RNA-seq data from biological duplicates were used to determine differential gene expression ([FDR] < 0.05). Right, heatmap showing expression levels of 145 2C genes ([FDR] < 0.05) in high and low ΔΨ_m_ ESCs. See Supplementary Data 1 for the 2C gene list. 2C, 2-cell. **c** MA plots from RNA-seq data showing up- (red) and down-regulated (blue) repetitive elements ([FDR] < 0.05) in low vs. high ΔΨ_m_ ESCs. Selected repetitive elements were highlighted. **d** RT-qPCR validation for upregulation of *MERVL* and 2C genes in low ΔΨ_m_ ESCs. Relative mRNA expression was normalized against that in high ΔΨ_m_ ESCs, which was arbitrarily set 1. Data were presented as mean ± SEM from four independent FACS sorting. *P* values were calculated using the two-sided Student’s *t* test. **e** A representative FACS plot from three biological replicates showing TMRM staining in naive ESCs treated with DMSO or CCCP. **f** RT-qPCR analysis for *MERVL* and 2C genes in naive ESCs treated with DMSO or CCCP. Relative mRNA expression was normalized against that in the CCCP-treated naive ESCs, which was arbitrarily set 1. Data were presented as mean ± SEM from three biological replicates. *P* values were calculated from the two-sided Student’s *t* test. **g** Unsupervised clustering using transcriptome of high and low ΔΨ_m_ ESCs, reported 2-cell-like cells and developmental stages of mouse embryogenesis. Green-shaded area denotes the close transcriptome correlation of low ΔΨ_m_ qESCs with other datasets. **h** Gene ontology (GO) analysis for genes significantly upregulated in low ΔΨ_m_ qESCs. GO terms with fold enrichment > 2 and the *P* value < 0.05 (defined by the Benjamini-Hochberg method) were considered as significant enrichment. Source data are provided as a Source Data file.

The spontaneous qESCs can be isolated by live-cell sorting after staining with the lipophilic cationic dye Tetramethylrhodamine Methyl Ester (TMRM)^5,15^ (Supplementary Fig. 1), which measures the MMP across the inner mitochondrial membrane. The MMP is a direct indicator of mitochondrial function and integrity^16,17^. Naive ESCs display intrinsic heterogeneity in MMP, demonstrated by both TMRM staining (Supplementary Fig. 2a, top) and the mitochondrial redox-sensing Grx1-roGFP2 reporter^18^ (Supplementary Fig. 2b). Naive ESCs with low MMP signals (bottom 5%, Supplementary Fig. 2a, bottom) exhibited typical features of quiescence^2,5^, including the low level of total RNAs per cell, an extremely slow proliferation rate and an increase of G_0_ arrest (Fig. 1a, Supplementary Fig. 2c–f). Like cycling ESCs, the spontaneous qESCs (low ΔΨ_m_/MMP) retained their ability to differentiate into three embryonic germ layers (Supplementary Fig. 2g, h).

Total RNA sequencing (RNA-seq) identified 1641 genes with altered expression in the spontaneous qESCs as compared to the ESCs with top 5% MMP (high ΔΨ_m_/MMP) after normalization against the ERCC spike-in control (false discovery rate [FDR] < 0.05) (Fig. 1b; Supplementary Fig. 3a, b and Supplementary Data 1). They included 1238 and 403 up- or down-regulated genes in the spontaneous qESCs, respectively. Expression of cell cycle genes (e.g., *Cdk1, Ccnb1, Ccnd3, Pdk1*) was significantly downregulated in the qESCs as expected (Fig. 1b, left and Supplementary Fig. 3c). While the pluripotency genes (e.g., *Oct4, Nanog, Sox2*) were expressed at similar levels in the quiescent and cycling ESCs (Supplementary Fig. 3d), endogenous retroviruses (e.g., *MERVL*, *RLTR20C, RLTR6B_Mm, ETnERV3-int*) and 2C-specific genes (e.g., *Zscan4*, *Dux*, *Gm4340*)^19^ were expressed at significantly elevated levels in the qESCs (Fig. 1b, c, Supplementary Fig. 3e and Supplementary Data 1). The RNA-seq results for the 2C gene signatures^19^ were validated by reverse-transcription and real-time PCR (RT-qPCR) (Fig. 1d). Importantly, increased expression of *MERVL* and 2C genes was also observed in naive ESCs treated with Carbonyl Cyanide 3-chlorophenylhydrazone (CCCP) or complex I electron transport chain inhibitor Phenformin, both of which reduce the MMP^20,21^ (Fig. 1e, f and Supplementary Fig. 3f, g). Elevated expression of 2C genes in the spontaneous qESCs was confirmed in two independent naive ESC lines, Oct4-GiP^22^ and V6.5 2i/LIF ESCs (Supplementary Fig. 3h). Consistently, the spontaneous qESCs isolated from the MERVL-2C::EGFP^23^ and Oct4-GiP naive ESC lines had higher levels of 2C::EGFP reporter signals and lower levels of OCT4-GFP signals, respectively (Supplementary Fig. 4a, b). ESCs cultured under the serum/LIF condition do not enter quiescence when mitochondrial function is blocked^5,24^ and therefore, did not show changes in 2C gene expression (Supplementary Fig. 3h).

Hierarchical clustering analysis showed that transcriptome of the spontaneous qESCs closely correlated with that of early-stage blastomeres (e.g., zygote, early 2C, 2C)^25^ as well as transient or genetically induced 2-cell-like cells (2CLCs) with expanded pluripotency^19,26–33^ (Fig. 1g, green-shaded area). Interestingly, the spontaneous qESCs were more closely associated with zygote and early 2C embryos as compared to the transient *MERVL*-tdTomato^+^ 2CLCs^19^ (Fig. 1g). Notably, the extraembryonic placenta development pathways were among the top ranked gene ontology (GO) terms upregulated in the spontaneous qESCs (Fig. 1h and Supplementary Data 1). As expected, transcriptome of the cycling ESCs (high ΔΨ_m_/MMP) resembled that of the inner cell mass and 2C-negative cells (e.g., Zscan4^low^, *MERVL*-tdTomato^-^) (Fig. 1g, unshaded area).

### The spontaneous qESCs exhibit unrestricted developmental potential

**Fig. 2:**
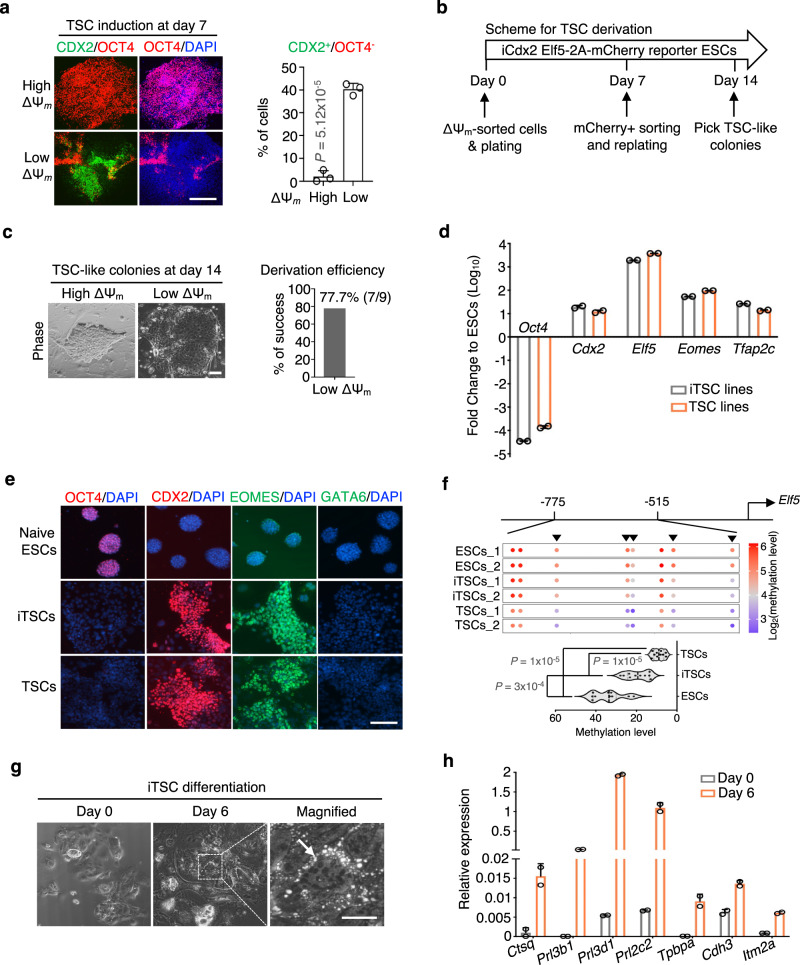
The spontaneous qESCs give rise to extraembryonic trophoblast stem cells. **a** Left, immunofluorescence of OCT4 (red), CDX2 (green) and nuclei (DAPI blue) at day 7 post TSC differentiation. Scale bar, 200 µm. Right, quantification of CDX2^+^/OCT4^−^ cells. Data were presented as mean ± SEM from three independent experiments. *P* values were calculated using the two-sided Student’s *t* test. TSC trophoblast stem cell. **b** Scheme for TSC derivation. **c** Left, representative images of typical TSC-like colonies at day 14. The TSC-like colonies were only observed from low ΔΨ_m_ ESCs, but not from high ΔΨ_m_ ESCs. Scale bar, 200 µm. Right, efficiency of TSC derivation. The success rate was calculated by dividing the number of successfully derived iTSC lines from total number of picked colonies. **d** RT-qPCR analysis for indicated genes in ESCs, TSCs and iTSCs. Relative mRNA expression was normalized against that in naive ESCs. Data from two independent cell lines were presented as mean ± SEM in the form of Log_10_ (fold change). **e** Immunofluorescence of indicated proteins in indicated cell types. Nuclei were co-stained with DAPI (blue). Scale bar, 200 µm. Images were representatives from two independent experiments. **f** Top, heatmap showing DNA methylation level at the upstream region of *Elf5* promoter. The heatmap score (Log_2_) was shown on the right. Each dot indicates a methylated CpG site. Dark arrowheads denote five CpG sites that exhibit substantial differences in methylation levels between ESCs and TSCs. Bottom, quantification of methylation level for five CpG sites shown from the top. Violin plots show the median, 1st and 3rd quartiles. Data from two independent cell lines were presented. *P* values were determined by a two-tailed, nonparametric Mann-Whitney test. **g** Representative images from two independent iTSC lines at day 6 of differentiation. The white arrow indicates the trophoblast giant cells. Scale bar, 200 µm. **h** RT-qPCR analysis for indicated genes in the iTSCs at day 0 and 6 of differentiation. Relative mRNA expression was normalized against that of *β-actin*. Data from two independent iTSC lines were presented as mean ± SEM. Source data are provided as a Source Data file.

The transcriptomic similarity between the spontaneous qESCs and 2C embryos suggests that the spontaneous qESCs may have unrestricted developmental potential. To test this, we subjected the cycling ESCs and qESCs to trophoblast stem cell (TSC) differentiation in vitro. The E14 qESCs exhibited a very high TSC induction efficiency, as denoted by around 40% of CDX2^+^/OCT4^−^ cells at day 7 post TSC induction, whereas the cycling ESCs still maintained robust OCT4 expression with little to no expression of trophoblast marker CDX2 (Fig. 2a). The qESCs expressing the iCdx2 Elf5-2A-mCherry reporter^34^ gave rise to stable TSC lines with ~77% efficiency (Fig. 2b, c) (referred to as iTSCs herein). The iTSCs demonstrated robust proliferation and exhibited expression levels of the canonical TSC markers *Cdx2, Elf5, Eomes*, *and Tfap2c* comparable to those derived directly from blastocysts (Fig. 2d). Similar to TSCs, the iTSCs also lacked expression of the pluripotent gene *Oct4* (Fig. 2d). Immunofluorescence analysis confirmed elevated levels of CDX2 and EOMES proteins in these cells, while OCT4 or the primitive endoderm marker GATA6 were not detected (Fig. 2e and Supplementary Fig. 4c). Furthermore, the CpG sites within the *Elf5* promoter were hypomethylated in iTSCs when compared to ESCs^35^ (Fig. 2f). Upon differentiation, the iTSCs underwent morphological alterations, characterized by enlarged and flattened cells, along with the emergence of trophoblast giant cells (TGCs) (Fig. 2g). Furthermore, iTSCs demonstrated the capability to differentiate into all subtypes of trophoblast lineages. Gene markers for the terminally differentiated TGCs, spongiotrophoblasts and labyrinthine trophoblasts, such as *Ctsq, Prl3b1, Prl3d1, Prl2c2, Tpbpa, Cdh3*, and *Itm2a*, were significantly upregulated (Fig. 2h). These data strongly support that the spontaneous qESCs have unrestricted cell fate in vitro.

### The spontaneous qESCs have distinct cellular metabolism

**Fig. 3:**
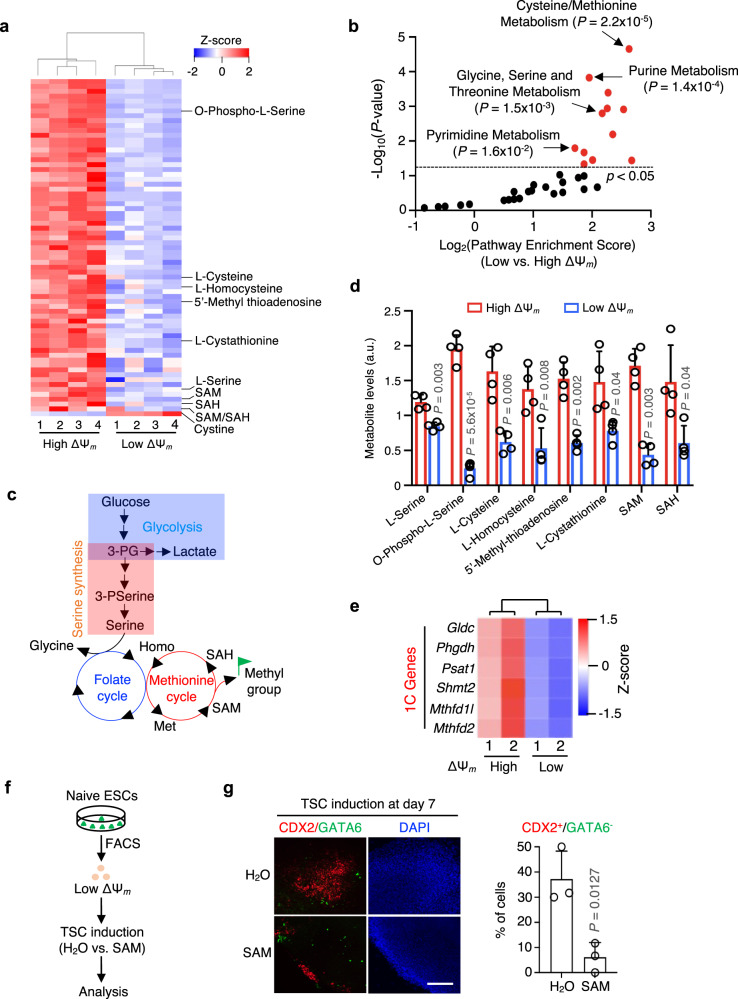
Low one-carbon metabolism confers unrestricted cell fate of the spontaneous qESCs. **a** Heatmap (Z-score) showing significantly changed metabolites in high and low ΔΨ_m_ ESCs. Metabolites with *P* < 0.05 were selected. Data from four biological replicates were presented as mean ± SEM. *P* values were determined by the two-sided Student’s *t* test. Metabolites, except Cystine, involved in one-carbon (1C) metabolism were highlighted on the right. **b** Pathway analysis for metabolites significantly decreased in the low ΔΨ_m_ qESCs. *P* < 0.05, determined by the Fisher’s Exact Test, was used as the cutoff for significantly enriched pathways. Selected pathways were highlighted. **c** An overview of 1C metabolism. 3-PG 3-phosphoglycerate, Homo homocysteine, Met methionine, SAM *S*-adenosylmethionine, SAH *S*-adenosylhomocysteine. **d** Levels of 1C-related metabolites as indicated at the bottom. Data were presented as mean ± SEM from four biological replicates. a.u. arbitrary unit. *P* values were determined by the two-sided Student’s *t* test. **e** Heatmap (Z-score) showing expression levels of 1C-related genes in high and low ΔΨ_m_ ESCs. RNA-seq data from biological duplicates with [FDR] < 0.05 were shown. **f** Scheme for TSC induction in the low ΔΨ_m_ qESCs treated with H_2_O or SAM. **g** Left, immunofluorescence of CDX2 (red), GATA6 (green) and nuclei (DAPI blue) at day 7 post TSC differentiation. Scale bar, 200 µm. Right, quantification of CDX2^+^/GATA6^−^ cells for data shown from the left. *P* values were calculated using the two-sided Student’s *t* test from three independent experiments. Source data are provided as a Source Data file.

We performed untargeted metabolomics analysis for naive ESCs with bottom or top 5% MMP (Fig. 3a). A total of 68 metabolites consistently displayed significant reductions in the qESCs across four independent experiments, with cystine being the sole metabolite showing a notable increase (Fig. 3a and Supplementary Data 2). Specifically, the qESCs demonstrated decreased levels of ATP and metabolites associated with the glycolysis, TCA cycle, pentose phosphate pathway, purine and pyrimidine metabolism (Fig. 3b, Supplementary Fig. 5a–c and Supplementary Data 2). Particularly striking was the marked downregulation of metabolites linked to one-carbon (1C) metabolism in the qESCs (Fig. 3a–c). The 1C metabolic pathway (encompassing folate and methionine cycles) recycles 1C units from glucose, vitamins and amino acid catabolism or through de novo synthesis via serine synthesis pathway (SSP) to fuel important cellular processes^36^ (Fig. 3c). As shown in Fig. 3b, the glycine, serine, threonine and methionine metabolic pathways were all significantly downregulated in the qESCs. Consistently, the levels of 1C metabolites such as L-homocysteine, SAM, and SAH, as well as SPP metabolites L-Serine and O-Phospho-L-Serine were notably reduced in the qESCs compared to the control cells (Fig. 3d). The metabolic changes in the qESCs were consistent with diminished expression of key genes in the 1C metabolic pathway (Fig. 3e), including *Gldc, Phgdh, Psat1, Shmt2, Mthfd1l*, and *Mthfd2*^36^.

SAM supplement is widely used to reverse low levels of intracellular SAM^37,38^. Upon SAM supplementation (Fig. 3f), the efficiency of iTSC induction decreased sixfold, as evidenced by the percentage of CDX2^+^/GATA6^−^ cells at day 7 post-induction (Fig. 3g). SAM supplementation also resulted in a global shift of the transcriptome from that of qESCs/2CLCs to conventional ESCs (see below). These results suggest that lower level of 1C metabolite SAM may contribute to increased cell fate plasticity in the qESCs.

### The spontaneous qESCs have global reduction of H3K27me3 and higher chromatin accessibility at genes important for the extraembryonic development

The 1C metabolite SAM is a universal methyl donor for histone/DNA methylases^39^. To examine whether the qESCs have a distinct epigenetic landscape, we assessed chromatin accessibility in qESCs by performing the Assay for Transposase Accessible Chromatin with high-throughput sequencing (ATAC-seq) (Supplementary Data 3). Chromatin accessibility was modestly reduced at 8768 gene loci in the qESCs, many of which were involved in development of three embryonic germ layers (Supplementary Fig. 6a, b, bottom). Interestingly, increase of chromatin accessibility was found at 2183 chromatin loci in the qESCs, which were enriched for genes important for extraembryonic development (Supplementary Fig. 6a, b, top).

**Fig. 4:**
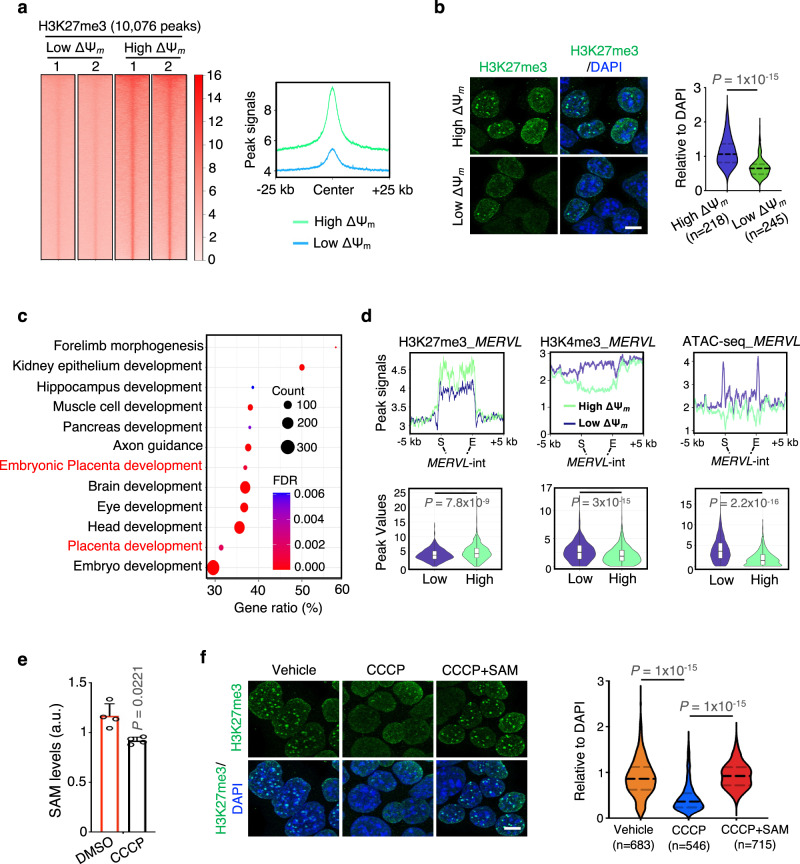
Global reduction of H3K27me3 enables unrestricted cell fate of the spontaneous qESCs. **a** Left, heatmaps of H3K27me3 CUT&RUN signals from biological duplicates. The number of differential peaks was shown on top. Right, line plots showing H3K27me3 peak signals from the left. **b** Left, immunofluorescence of H3K27me3 (green) and nuclei (DAPI blue) in high and low ΔΨ_m_ ESCs. Scale bar, 10 µm. Right, quantified H3K27me3 fluorescence signals of polycomb bodies (foci) relative to DAPI. Violin plots show the median, 1st and 3rd quartiles. Data from three biological replicates were presented. *P* values were determined by a two-tailed, nonparametric Mann-Whitney test. *n* number of foci. **c** GO analysis for H3K27me3 peaks that were decreased in the low ΔΨ_m_ qESCs. “Count” denotes the number of genes found in each GO term. **d** Top, line plots showing changes of H3K27me3, H3K4me3, and ATAC-seq signals at 662 *MERVL* elements in low and high ΔΨ_m_ ESCs. Bottom, violin box plots representing the peak values of the top plots show the median, 1st, 3rd quartiles, lower adjacent value, upper adjacent value, and outside points. *P* values were determined by a nonparametric Mann-Whitney test. **e** Levels of intracellular SAM in naive ESCs treated with DMSO or CCCP. Data were presented as mean ± SEM from four biological replicates. a.u. arbitrary unit. *P* values were determined by the two-sided Student’s *t* test. **f** Left, immunofluorescence of H3K27me3 (green) and nuclei (DAPI blue) at indicated conditions. Scale bar, 10 µm. Right, quantification of the left. Violin plots denote quantified H3K27me3 fluorescence signals of foci relative to DAPI. Data were from three biological replicates. *P* values were determined by a two-tailed, nonparametric Mann-Whitney test. *n* number of foci. Source data are provided as a Source Data file.

We performed Cleavage Under Targets and Release Using Nuclease (CUT&RUN) analysis for H3K4me3 and H3K27me3 marks, given their prominent functions in naive ESCs^40,41^. While expression of the core subunits (*Suz12, Eed, Ezh1, Ezh2*) of PRC2 complex^42^ remained unchanged (Supplementary Fig. 8a), there was a drastic global reduction of H3K27me3 in the qESCs (Fig. 4a and Supplementary Data 4). This was further confirmed by the immunofluorescence analysis (Fig. 4b). In contrast, the H3K4me3 level had no discernable difference in the qESCs (Supplementary Fig. 6c, d, and Supplementary Data 5). Reduction of H3K27me3 in the qESCs mostly occurred at intergenic or intron regions in the genome (Supplementary Fig. 7a). These regions were enriched for the consensus sequences for CDX2 and ELF5^43^ by Motif analysis (Supplementary Fig. 7b) and were associated with multi-tissue development, including the placenta development pathways (Fig. 4c). Representative H3K27me3 occupancy at *Cdx2* and *Tfap2c* was shown in Supplementary Fig. 7c.

We next examined chromatin changes at the long terminal repeat (LTR) sequences of endogenous retrovirus *MERVL* elements. The LTRs evolve as functional promoters to regulate transcription of nearby genes (e.g., 2C genes), leading to generation of chimeric transcripts in ESCs and early embryos^19,44^. Consistent with elevated expression of *MERVL* (Fig. 1c, d), H3K27me3 was significantly downregulated in 662 *MERVL* elements in the qESCs, with concomitant increase of H3K4me3 and chromatin accessibility (Fig. 4d). Epigenomic and transcriptomic features at a representative *MERVL* locus were shown in Supplementary Fig. 7d. In contrast, only modest changes in H3K27me3, H3K4me3 and ATAC-seq signals were found at other 2C gene loci in the qESCs (Supplementary Fig. 7e–g), suggesting a specific and non-redundant function of H3K27me3 in de-repressing *MERVL* expression in qESCs.

### CCCP treatment and MAT2A depletion lead to unrestricted cell fate in naive ESCs

To examine whether reducing MMP alters the metabolic and epigenetic states (Fig. 3a, d), we treated naive ESCs with CCCP for 9 h (Fig. 1e). Metabolomics analyses showed a decrease in the SAM level (Fig. 4e) while almost all TCA cycle metabolites, except alpha-ketoglutaric acid, remained unchanged in ESCs after CCCP treatment (Supplementary Fig. 8b and Supplementary Data 6). The CCCP treatment led to elevated expression of 2C gene signature, *MERVL* and the trophoblast master regulators (e.g., *Cdx2, Tfap2c, Eomes)* (Fig. 1e, f and Supplementary Fig. 8c). We also observed global reduction of H3K27me3, which was fully restored by SAM supplementation (Fig. 4f). H3K9me3 and DNA methylation marks were not affected by the CCCP treatment (Supplementary Fig. 8d, e).

**Fig. 5:**
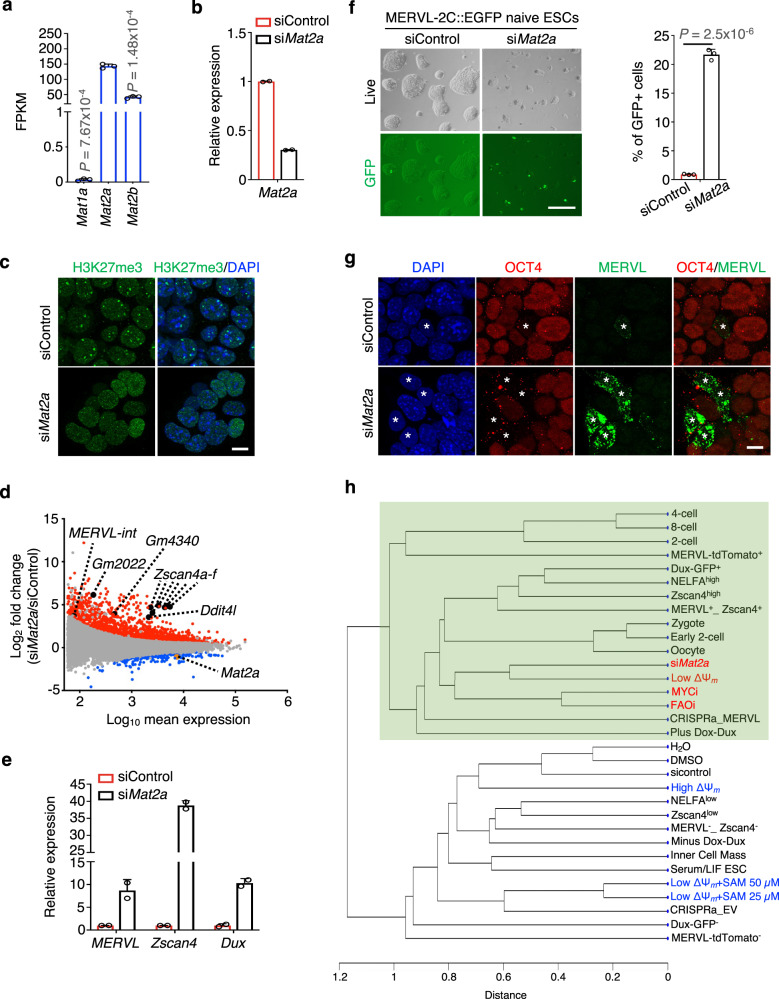
Knockdown of *Mat2a* induces 2C-like features in naive ESCs. **a** mRNA expression levels of *Mat1a, Mat2a and Mat2b* in naive ESCs. FPKM values were from RNA-seq data^7^. Data from three biological replicates were presented as mean ± SEM*. P* values were calculated using the two-sided Student’s *t* test. FPKM, fragments per kilobase of transcript per million. **b** RT-qPCR validation for *Mat2a* knockdown. Relative mRNA expression was normalized against that in the siControl naive ESCs, which was arbitrarily set 1. Data from two biological replicates were presented as mean ± SEM. **c** Confocal images of H3K27me3 (green) and nuclei (DAPI blue) immunofluorescence at indicated conditions. Scale bar, 10 µm. Representative images from two independent experiments were shown. **d** MA plots showing up- (red) and down-regulated (blue) genes in si*Mat2a* versus siControl naive ESCs. Selected 2C genes, *Mat2a*, and *MERVL* were highlighted. RNA-seq data from biological duplicates were used to determine differential gene expression ([FDR] < 0.05). **e** RT-qPCR validation for upregulated 2C gene signature in si*Mat2a* naive ESCs. Relative mRNA expression was normalized against that in siControl naive ESCs, which was arbitrarily set 1. Data were presented as mean ± SEM of two biological replicates. **f** Left, representative live and GFP images of MERVL-2C::EGFP naive ESCs at indicated conditions. Scale bar, 200 µm. Right, quantification of GFP + cells (%) for data shown from the left. Data from three independent experiments were presented as mean ± SEM. *P* values were calculated using the two-sided Student’s *t* test. **g** Immunofluorescence of OCT4 (red), MERVL (green) and nuclei (DAPI blue). Scale bar, 10 µm. Asterisks indicate MERVL^+^ cells that lose chromocenters and OCT4 protein. Note that chromocenters were marked by DAPI-dense regions in the MERVL^−^ cells, which become spread in the MERVL^+^ cells. Images were representatives from two independent experiments. **h** Unsupervised clustering using transcriptome data of indicated samples (see method). Green-shaded area denotes the close transcriptome correlation of samples highlighted in red with other datasets. Source data are provided as a Source Data file.

We next examined whether blocking SAM biosynthesis in naive ESCs affects H3K27me3 and de-repression of 2C gene signature. The methionine adenosyltransferase (MAT) family proteins, consisting of three isoforms MAT1A, MAT2A and MAT2B^45^, are the rate-limiting enzymes that catalyze SAM synthesis from methionine and ATP in mammals^45,46^. Among them, *Mat2a* was expressed at the highest level in naive ESCs (Fig. 5a). Knockdown of *Mat2a* by siRNA in naive ESCs resulted in global reduction of H3K27me3 levels (Fig. 5b, c). Notably, *Mat2a* knockdown significantly upregulated the expression of *MERVL* and 2C genes, as revealed by RNA-seq analysis and validated by RT-qPCR (Fig. 5d, e and Supplementary Data 7). Similarly, MERVL-2C::EGFP naive ESCs had over 20-fold increase in GFP signals after *Mat2a* knockdown (Fig. 5f). Additionally, the *MERVL*^*+*^ cells exhibited a loss of chromocenters, indicated by the absence of DAPI-dense regions, and reduced levels of OCT4 (Fig. 5g). RNA-seq analyses revealed a close correlation between the transcriptome of *Mat2a*-depleted naive ESCs and that of spontaneous qESCs and early-stage blastomeres (Fig. 5h), distinct from qESCs with SAM supplementation and conventional ESCs (Fig. 5h). These findings underscore the importance of low MMP and SAM homeostasis in regulating the fate plasticity of qESCs.

### Inducing quiescence in naive ESCs is sufficient to confer unrestricted cell fate

**Fig. 6:**
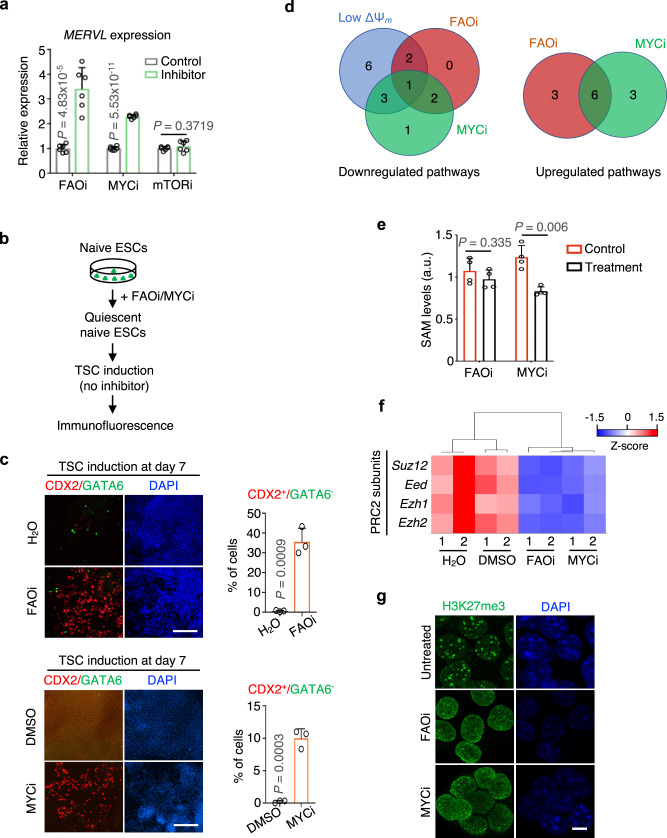
Induction of quiescence in naive ESCs is sufficient to unlock the unrestricted developmental potential via downregulating H3K27me3 and metabolic modulation. **a** RT-qPCR analysis for *MERVL* in naive ESCs treated with FAO, MYC or mTOR inhibitor (see method). Relative mRNA expression was normalized against that of the control cells, which was arbitrarily set 1. Data were presented as mean ± SEM from six biological replicates. *P* values were calculated using the two-sided Student’s *t* test. **b** Scheme for TSC induction in FAOi or MYCi induced quiescent naive ESCs. **c** Left, immunofluorescence of CDX2 (red), GATA6 (green) and nuclei (DAPI blue) at day 7 post TSC differentiation at indicated conditions. Scale bar, 200 µm for FAOi and 400 µm for MYCi. Right, quantification of CDX2^+^/GATA6^−^ cells for data shown from the left. Data were presented as mean ± SEM from three independent experiments. *P* values were determined by the two-sided Student’s *t* test from three independent experiments. **d** Venn diagram comparing downregulated (left) and upregulated (right) pathways for metabolites that showed significant changes in low ΔΨ_m_, FAOi and MYCi (see Supplementary Data 6 for more details). **e** Levels of intracellular SAM in naive ESCs treated with FAOi or MYCi as compared to control. Data were presented as mean ± SEM from four biological replicates. a.u. arbitrary unit. *P* values were determined by the two-sided Student’s *t* test. **f** Heatmap (Z-score) showing expression levels of the PRC2 core subunits in indicated conditions. RNA-seq data from biological duplicates were shown. **g** Confocal images of H3K27me3 (green) and nuclei (DAPI blue) immunofluorescence at indicated conditions. Scale bar, 10 µm. Representative images from three independent experiments were shown. Source data are provided as a Source Data file.

To examine whether inducing quiescence is sufficient to confer unrestricted cell fate, we performed RNA-seq analysis for the qESCs induced by FAO^5^ or MYC^6^ inhibitor (referred to as FAOi or MYCi herein). Hierarchical clustering analysis showed that the transcriptome of FAOi and MYCi-induced qESCs was closely clustered with that of early stage blastomeres (Fig. 5h), akin to the clustering pattern observed in the spontaneous qESCs and *Mat2a*-depleted naive ESCs. RT-qPCR analysis confirmed a significant increase in *MERVL* expression in the FAOi and MYCi-induced qESCs (Fig. 6a). In contrast, mTOR inhibition-mediated ESC growth arrest^7^ (Supplementary Fig. 9a, b), which does not induce quiescence, did not upregulate the *MERVL* expression (Fig. 6a). Both FAOi- or MYCi-induced qESCs exhibited efficient differentiation into TSCs, yielding a high percentage of CDX2^+^/GATA6^−^ cells by day 7 post-induction (Fig. 6b, c).

The untargeted metabolomics analyses conducted on the spontaneous and induced qESCs showed that FAOi or MYCi-induced qESCs had significantly lower levels of TCA metabolites, such as alpha-ketoglutaric acid, as well as metabolites within the purine and pyrimidine metabolism pathways (Fig. 6d, left, Supplementary Figs. 5c,9c, d and Supplementary Data 6), mirroring the metabolic profiles observed in spontaneous qESCs. However, FAOi or MYCi-induced qESCs also displayed significant upregulation in glycerolipid, pentose phosphate and glutathione metabolism pathways (Fig. 6d, right and Supplementary Data 6). Importantly, MYCi and to a lesser degree, FAOi, led to a decrease in SAM levels (Fig. 6e), downregulation of the PRC2 subunits (Fig. 6f) and reduction of global H3K27me3 (Fig. 6g). Together, these results suggest that inducing quiescence in naive ESCs results in a convergent change on H3K27me3.

### Genetic deletion of *Eed* promotes unrestricted ESC fate

**Fig. 7:**
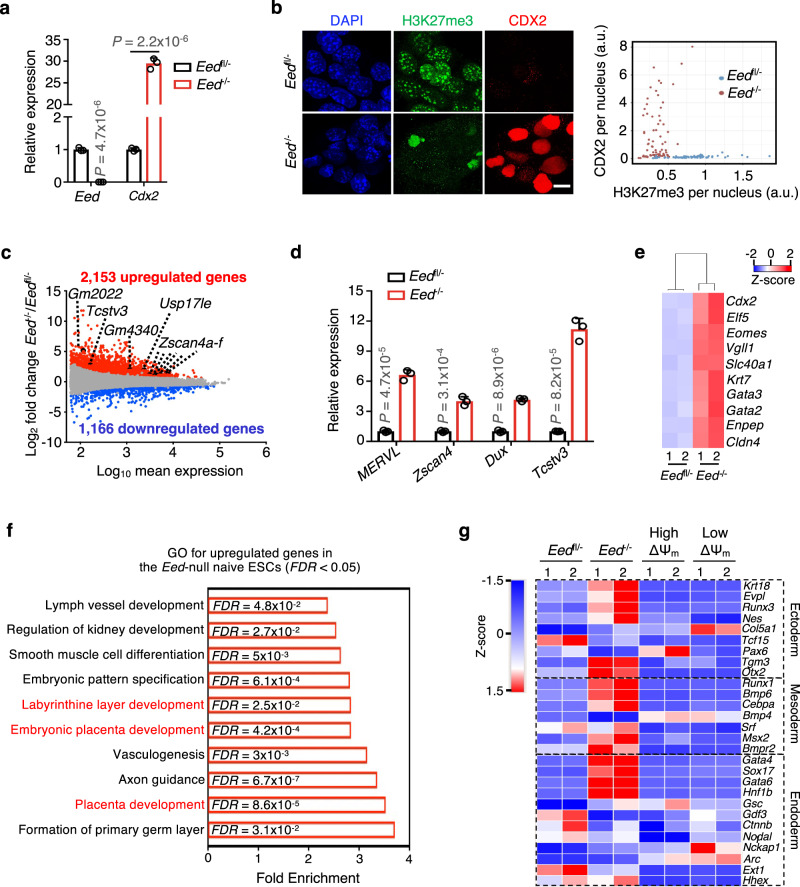
Deletion of *Eed* promotes unrestricted fate potential in naive ESCs. **a** RT-qPCR analysis for *Eed* and *Cdx2* in *Eed*^*fl/−*^ (heterozygous) and *Eed*^*−/−*^ (null) naive ESCs. Relative mRNA expression was normalized against that of the *Eed*^*fl/-*^ naive ESCs, which was arbitrarily set 1. Data were presented as mean ± SEM from three biological replicates. *P* values were calculated using the two-sided Student’s *t* test. **b** Left, confocal images of H3K27me3 (green), CDX2 (red) and nuclei (DAPI blue) immunofluorescence in the *Eed*^*fl/−*^ and *Eed*^*−/−*^ naive ESCs. Scale bar,10 µm. Right, scatter plot showing per-nucleus intensity of CDX2 and H3K27me3 shown from the left. A total of 145 nuclei (69 nuclei for *Eed*^*fl/-*^ and 76 nuclei for *Eed*^*−/−*^) from one representative experiment were shown. **c** MA plots showing up- (red) and down-regulated (blue) genes in the *Eed*^*−/−*^ naive ESCs relative to *Eed*^*fl/−*^ naive ESCs. 2C genes were selectively highlighted. RNA-seq data from biological duplicates were used to determine differential gene expression ([FDR] < 0.05). **d** RT-qPCR analysis showing elevated expression of *MERVL* and 2C genes in the *Eed*^*−/−*^ naive ESCs. Relative mRNA expression was normalized against that in *Eed*^*fl/-*^ naive ESCs, which was arbitrarily set 1. Data were presented as mean ± SEM of three biological replicates. *P* values were calculated by the two-sided Student’s *t* test. **e** Heatmap (Z-score) showing expression levels of trophectoderm genes in the *Eed*^*fl/−*^ and *Eed*^*−/−*^ naive ESCs. RNA-seq data from biological duplicates were shown. **f** GO analysis for genes significantly upregulated in the *Eed*^*−/−*^ naive ESCs. GO terms with fold enrichment > 2 and the [FDR] < 0.05 (defined by the Fisher’s Exact Test) were considered as significant enrichment. **g** Heatmap (Z-score) showing expression of genes associated with ectoderm, mesoderm and endoderm in indicated cell types. RNA-seq data from biological duplicates were shown. Source data are provided as a Source Data file.

Given the observed reduction of H3K27me3 in both spontaneous and induced qESCs, we investigated whether the genetic deletion of *Eed* (embryonic ectoderm development), the core subunit of the PRC2 complex^47^, could remove the developmental constraints in naive ESCs. To this end, we derived naive ESCs from the *Eed*^*−*^^*/−*^ (null) and *Eed*^*fl/−*^ (heterozygous) mouse embryos and maintained them continuously in 2i/LIF medium (Fig. 7a, b and Supplementary Fig. 10a, b, see method). The *Eed*^*−/−*^ naive ESCs exhibited global loss of H3K27me3 (Fig. 7b). RNA-seq analysis identified 2153 upregulated and 1166 downregulated genes ([FDR] < 0.05) in the *Eed*^*−/−*^ naive ESCs (Fig. 7c). Similar to spontaneous qESCs and *Mat2a*-depleted naive ESCs, the *Eed*^*−/−*^ naive ESCs had elevated expression of *MERVL and* 2C genes (such as *Zscan4, Tcstv3 Gm2022)* (Fig. 7c, d and Supplementary Data 7). Genes from both embryonic and extraembryonic differentiation pathways were upregulated in the *Eed*^*−/−*^ naive ESCs (Fig. 7e–g). Strikingly, the *Eed*^*−/−*^ naive ESCs had a significant increase in expression of trophectoderm gene markers (e.g., *Cdx2, Elf5, Eomes*), even without TSC induction (Fig. 7a, b, e). These results imply that the *Eed*^*−/−*^ naive ESCs are different from the spontaneous qESCs, which had minimal changes in expression of ectoderm, mesoderm, and endoderm genes (Fig. 7g).

### Quiescence endows a dynamic reservoir of naive ESCs with the potential for unrestricted cell fate

**Fig. 8:**
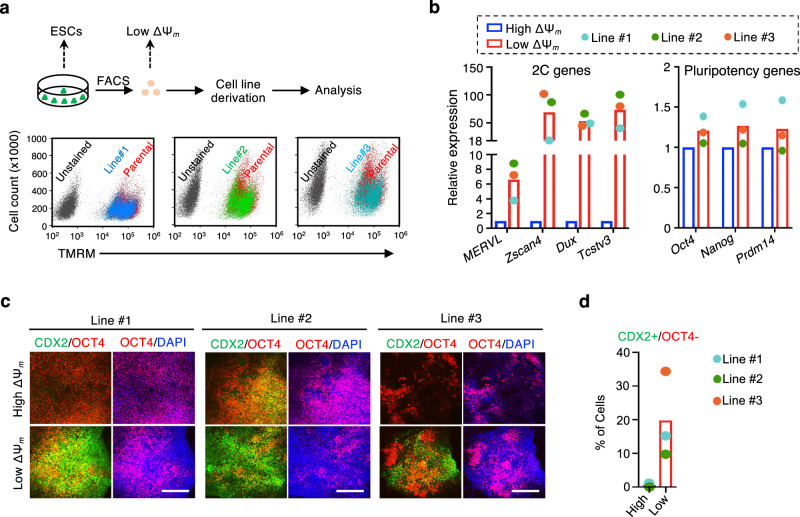
Quiescence preserves innate unrestricted cell fate in naive ESCs. **a** Top, scheme for generating new naive ESC lines from the low ΔΨ_m_ single qESC. Bottom, TMRM staining showing the similarity of ΔΨ_m_ heterogeneity between parental cells and three newly established naive ESC lines. **b** RT-qPCR analysis for genes related to 2C (left) and pluripotency (right) in high and low ΔΨ_m_ ESCs isolated from three single cell derived naive ESC lines. Relative mRNA expression was normalized against that in the high ΔΨ_m_ ESCs, which was arbitrarily set 1. Average values were presented from three single cell derived naive ESC lines. **c** Left, immunofluorescence of OCT4 (red), CDX2 (green) and nuclei (DAPI blue) for TSC induction at day 7 from three single cell derived naive ESC lines. Scale bar, 200 µm. Representative images from three independent experiments for each cell lines were shown**. d** Quantification of CDX2^+^/OCT4^−^ cells for data shown in (**c**). Average values were presented from three single cell derived naive ESC lines. Source data are provided as a Source Data file.

Unlike adult stem cells, which predominantly remain quiescent within their niche in vivo, naive ESCs continuously proliferate in vitro. We postulated that quiescence enables naive ESCs to sustain a dynamic reservoir of cells with unrestricted cell fate. Mathematical modeling through simulation supported the notion that stochastic mitochondrial flux in daughter cells during cell division can sustain metabolic heterogeneity in naive ESCs (Supplementary Fig. 11a, b). In line with the modeling predictions, isolated single spontaneous qESCs were capable of generating ESC lines with similar heterogeneity in MMP as the parental cell line (Fig. 8a). Importantly, the low MMP qESCs derived from each newly established naive ESC line displayed unrestricted cell fate. They expressed high levels of *MERVL* and 2C genes (*Zscan4, Dux, Tcstv3*) (Fig. 8b, left) as well as pluripotency markers *Oct4, Nanog*, and *Prdm14* (Fig. 8b, right). These cells efficiently differentiated into TSCs, as evidenced by a high percentage of CDX2^+^/OCT4^−^ cells at day 7 post-induction (Fig. 8c, d). These results suggest that quiescence enables maintenance of the intrinsic unrestricted cell fate in naive ESCs.

## Discussion

Quiescent stem cells are widely considered to reside in a “dormant” or “idle” state^1,2^. Here we report that spontaneous or induced quiescent ESCs represent a dynamic reservoir of cells endowed with unrestricted differentiation potential. These qESCs exhibit convergent alterations in mitochondrial activity, decreased levels of SAM, and global reduction of H3K27me3. These metabolic and epigenetic alterations accompany the drastic transcriptomic and functional changes in qESCs, including de-repression of the 2C and extraembryonic gene programs, and expanded differentiation potential towards the extraembryonic lineages. Our results strongly argue that stem cell quiescence transcends a mere dormant or inactive state. Instead, the qESCs actively acquire new distinctive metabolic and epigenetic states, enabling unrestricted cell fate determination. Our findings underscore a previously overlooked paradigm wherein quiescence serves as an active strategy employed by the stem cells to maximize cell fate plasticity.

We show that qESCs have convergent changes in PRC2-mediated H3K27me3 along with removal of developmental constraints. Unlike serum/LIF ESCs^40,48^, naive mouse ESCs are devoid of DNA methylation and mostly rely on H3K27me3 to repress lineage-specific gene expression^41,49^. Our study extends the function of PRC2 to direct regulation of endogenous retroviruses, 2C and TSC genes (e.g., *Cdx2, Elf5, Eomes*). Our results contrast to a recent report^41^ indicating that *Eed* deletion in mouse ESCs leads to minimal changes in the expression of TSC genes (Supplementary Data 7). The discrepancy might stem from the method employed in the prior study^41,50^, where *Eed* deletion occurred in serum/LIF ESCs prior to their transfer to 2i/LIF medium. Given that inhibition of PRC2 in naive human ESCs (hESCs) sporadically induces the trophoblast marker GATA3 in about 10% of cells^51,52^, it is plausible that PRC2 plays a conserved role in suppressing extraembryonic cell fate. Future investigations are warranted to explore whether quiescence in hESCs^5^ can expand cell fate and if so, whether it is through modulating PRC2-mediated H3K27me3.

Our study suggests importance of SAM homeostasis in naive ESCs. Cellular metabolites such as SAM, acetyl-CoA and α-ketoglutarate are substrates or cofactors for DNA- and histone-modifying enzymes and may contribute to regulation of the epigenetic landscapes in cells^16,53^. We find that both spontaneous and induced qESCs have lower levels of intracellular SAM. Acute reduction of MMP by CCCP also lead to reduced SAM levels in naive ESCs. Previous studies have indicated that the mitochondrial polarity is essential for transporting charged molecules in and out of mitochondria^17^. How MMP modulates SAM homeostasis in naive ESCs remains to be determined. Nonetheless, we find that blocking SAM biosynthesis by depletion of *Mat2a* is sufficient to induce the unrestricted cell fate. Conversely, SAM supplementation in low MMP ESCs can restrict extraembryonic differentiation. Collectively, these results suggest a potential connection between MMP state and SAM biosynthesis in regulating developmental potential of naive ESCs, which awaits further investigations.

One surprising finding is that low levels of SAM in qESCs correspond to a prominent reduction of global H3K27me3, along with minimal changes in H3K4me3, H3K9me3 or DNA methylation. The mechanism for such specific, rather than general, changes in chromatin modifications remains unclear. Specific regulation of H3K27me3 by intracellular SAM has been reported in naive hESCs^54^. The nonlinear correlation between the cellular SAM level and H3K27me3 could be influenced by various factors, including the overall abundance of enzymes and their antagonists (e.g., demethylases), as well as adaptive epigenetic mechanisms in response to SAM depletion^55^. These aspects merit further investigations in future studies. In contrast to H3K27me3, which emerges as a convergent change in both spontaneous and induced qESCs, the metabolic alterations in these cell types are largely distinct. Induced qESCs exhibit significantly elevated metabolites associated with pyrimidine metabolism and pentose phosphate pathway (Fig. 6d, right and Supplementary Data 6) that were not observed in the spontaneous qESCs. The functional significance of these pathways in cell fate determination awaits further studies.

Finally, our study shows that ESC quiescence can serve as a valuable platform for comprehensive metabolomics and epigenomic characterizations associated with cell fate alteration, which are somewhat limited for the in vivo quiescent adult or cancer stem cells. Bona fide quiescent ESCs can be isolated by FACS after TMRM live stain or stably induced using a single chemical agent (i.e., FAOi or MYCi), offering a more accessible and well-controlled experimental setting. Leveraging qESCs enables the establishment of fundamental principles related to cell metabolism, cell cycle regulation, and epigenetic modifications in stem cell fate determination.

## Methods

### Ethical regulation compliance

The 3–8-week-old female and 2–6-month-old male mice were used for all experiments. Mice were generated and maintained on the 129/S1 background on a 12 h light/dark cycle in the Transgenic Animal Model Core, University of Michigan. All animal procedures were conducted according to the protocols approved by the University Committee on Use and Care of Animals at the University of Michigan (protocol #PRO00004007 and PRO00006455).

### Cell lines and cell culture conditions

Mouse ES cell lines (ESCs) used in this study include: E14tg2a (E14) (ATCC, #CRL-1821TM), Oct4-GiP ESCs^22^, male V6.5 ESCs (Novus Biologicals, #NBP1-41162) and iCdx2 Elf5-2A-mCherry reporter ESCs^34^. All ESCs were routinely cultured on tissue culture plates coated with Geltrex (Thermo Fisher Scientific, #A1413202, 1:100 dilution from stock) at 37 ^o^C and 5% CO_2_. Naive ESCs were maintained under 2i/LIF culture condition, which contains N2B27 basal medium supplemented with PD0325901 (SIGMA ALDRICH, #PZ0162, 1 µM), CHIR99021 (SIGMA ALDRICH, #SML1046, 3 µM) and LIF (Millipore, #ESG1106, 1000 U/ml). N2B27 consists of a 1:1 mix of DMEM/F12 (Thermo Fisher Scientific, #21331-046) and Neurobasal (Thermo Fisher Scientific, #21103-049), N2 (Thermo Fisher Scientific, #17502001, 1:100), B27 (Thermo Fisher Scientific, #17504-044, 1:100), 2 mM Glutamine (Thermo Fisher Scientific, #25030024), Penicillin-Streptomycin (Thermo Fisher Scientific, #15140122, 1:100) and 0.055 mM 2-mercaptoethanol (Thermo Fisher Scientific, #21985023). Cells were routinely plated at the density of 1 × 10^5^ cells per well on the six-well tissue culture plates. Cells were passaged every 2 days by dissociation with StemPro Accutase (Gibco, #A1110501). Where indicated, ESCs were grown in serum/LIF culture condition containing the KnockOut™ DMEM medium (Thermo Fisher Scientific, #10829018) supplemented with 15% fetal bovine serum (FBS) (SIGMA ALDRICH, #F0926), 2 mM Glutamine, 1X Non-essential amino acids (Thermo Fisher Scientific, #11140050), 0.055 mM 2-mercaptoethanol, Penicillin-Streptomycin (1:100) and LIF (1000 U/ml).

Mouse embryonic fibroblasts (MEFs) were isolated from E13.5 embryos of FVB triple transgenic mice at the University of Michigan Transgenic Animal Model Core. Mitomycin-C-treated MEFs were maintained in KnockOut™ DMEM medium containing 15% FBS, 2 mM Glutamine, 1X Non-essential amino acids, Penicillin-Streptomycin (1:100) and 0.055 mM 2-mercaptoethanol. MEFs were prepared at the appropriate density (see below) and used within one week after plating.

TSCs were derived directly from Ẻ.3.5 blastocyst as previously described^56^. TSCs were routinely maintained on top of MEFs (30,000 cells per cm^2^) at 37 ^o^C and 5% CO_2_ in the TSC medium containing 70% MEF-conditioned media (R&D systems, #AR005), 30% base medium (RPMI 1640, Thermo Fisher Scientific, #11875093; sodium pyruvate, Thermo Fisher Scientific, #11360070, 1:100; Penicillin-Streptomycin, 1:100; 0.1 mM 2-mercaptoethanol and 20% FBS), 25 ng/ml FGF4 (Reprotech, #100-31) and 1 µg/ml Heparin (SIGMA ALDRICH, #H3149-10KU). Medium change was performed daily, and cells were passaged by dissociation with Trypsin-EDTA 0.05% (Thermo Fisher Scientific, #25300054).

### Derivation of *Eed*-null naive ESCs

Mouse strain JF1 carries a conditional mutation of *Eed* gene in which its exon 7 was flanked by *loxP* sequences. The *Eed*^*fl/fl*^ mice were generated and maintained on the 129/S1 background as described^47^. E3.5 embryos were obtained from a cross between *Eed* heterozygous females and *Eed* heterozygous males carrying Protamine CRE recombinase. Each E3.5 blastocyst was placed on top of MEF feeder in 2i/LIF culture condition containing the KnockOut™ DMEM base medium, 15% KnockOut™ Serum Replacement (Thermo Fisher #10828028), 5% FBS, 2 mM Glutamine, 1X Non-essential amino acids, 0.055 mM 2-mercaptoethanol and Penicillin-Streptomycin (1:100), PD0325901 (1 µM), CHIR99021 (3 µM) and LIF. At 4–5 days post plating, each inner cell mass was picked, dissociated into single cells and expanded on MEF feeders in the 2i/LIF ESC medium.

### Derivation of ESC lines from single low ΔΨ_m_ qESC

Each single naive ESC with bottom 5% TMRM fluorescence intensity was FACS-sorted directly onto each well of 96-well tissue culture plate. After 5–7 days, each colony was dissociated into single cell by StemPro Accutase, resuspended into 2i/LIF medium and transferred to 48-well tissue culture plates. Cell lines were routinely cultured in 2i/LIF medium.

### Generation of MERVL-2C::EGFP naive ESC lines

About 3 × 10^4^ E14 naive ESCs were seeded on 24-well plates 24 h before transfection. Cells were transfected with the 2C-EGFP plasmid (addgene, #69071)^23^ for 2 h by Lipofectamine^TM^ 2000 (Thermo Fisher Scientific, #11668027). After transfection, fresh medium was added overnight. Transfected cells were dissociated and replated at clonal density in 6-well tissue culture plates. Selection was performed with 250 µg/ml G418 (Thermo Fisher Scientific, #10131035) for one week before individual colonies were manually picked and expanded in 2i/LIF condition.

### Generation of mito-Grx1-roGFP2 naive ESC lines and live-cell imaging

The pPBmitoCOX8-Grx1-roGFP2 was generated by PCR amplification of mitoCOX8-Grx1-roGFP2 cassette using the Forward-GGAAGATCTATGTCCGTCCTGACGCCGC; Reverse-CGACGCGTTAACGTTCGAGGTCGACTC primers. PCR products were cloned into BglII/MluI sites in pPBhCMV1cHApA. E14 naive ESCs (2i/LIF) were transfected with 0.5 µg of pPBCAG-rtTAM2-IN, 0.5 µg of pPBmitoCOX8-Grx1-roGFP2 and 1 µg pCAG-hyPBase transposase by Lipofectamine^TM^ 2000. Individual colonies were manually picked and expanded under 2i/LIF condition after one-week selection with 250 µg/ml G418. Expression of mitoCOX8-Grx1-roGFP2 in the mitochondria (i.e., mito-Grx1-roGFP2 ESCs) was induced by Dox (1 µg/ml) and analyzed on a Nikon X1 Yokogawa Spinning Disk Confocal (Nikon Instruments Inc.). The 60× objective (water immersion) was used for live-cell imaging. The mito-Grx1-roGFP2 sensor was excited by the 405 nm and 488 nm laser line and detected the single emission (~520) through 500–554 nm bandpass filter.

### Generation of induced TSC lines and in vitro differentiation

The naive ESC line with the iCdx2 Elf5-2A-mCherry reporter^34^ was used to generate induced TSC lines (iTSC). About 400 FACS-sorted cells were plated directly onto MEF feeder (10,000 cells per cm^2^) in TSC medium. At day 7, mCherry+ cells were FACS-sorted and plated at clonal density for 7 days before individual TSC-like colonies were picked and expanded. The iTSC lines were routinely cultured at high density on MEF feeders (30,000 cells per cm^2^). To induce differentiation, the iTSCs (after MEF removal) were cultured on gelatin-coated dishes in the absence of FGF4 and Heparin for 6 days before analyses.

### CCCP and phenformin treatment of naive ESCs

To inhibit MMP, carbonyl cyanide 3-chlorophenylhydrazone (CCCP, SIGMA ALDRICH, #C2759, 3 µM) was added 24 h after cell plating. All analyses were performed 9 h after CCCP treatment. To inhibit complex I electron transport chain, naive ESCs were treated with phenformin (Selleck Chemical, #S2542, 1 mM) for 2 days. For *S*-(5′-Adenosyl)-L-methionine chloride dihydrochloride (SIGMA ALDRICH, #A7007, 0.5 mM) supplement, naive ESCs were plated on Lab-Tek II Chamber Coverglass (Thermo Fisher Scientific, #155382). At 24 h post plating, cells were pre-treated with 0.5 mM of SAM or vehicle for 2 h before treatment with CCCP for 6 h. SAM at a final concetration of 25 or 50 µM was used for culturing the spontaneous qESCs for 24 h.

### Induction of quiescence

Naive ESCs were induced to achieve a stable quiescent state by inhibition FAO or MYC (FAOi or MYCi). To inhibit FAO, Etomoxir (SIGMA ALDRICH, #E1905, 100 µM) was added at the time of plating for 4 days^5^. 10058-F4 (SIGMA ALDRICH, #475956) was used at a final concentration of 64 µM for 3 days to inhibit MYC^6^. As a control, naive ESCs were treated with mTOR inhibitor (mTORi) INK128 (Medchem Express, HY13328, 200 nM) for 3 days as described^7^. H_2_O and DMSO were added at the same final volume as FAOi, MYCi or mTORi. The induced qESCs and control cells were then harvested for RT-qPCR, metabolomics analysis and TSC differentiation.

### Mitochondrial membrane potential (ΔΨ_m_) staining and FACS sorting

Fractionation of ESCs with high and low ΔΨ_m_ levels was performed as previously described^5,15^. 25 nM of TMRM (Thermo Fisher Scientific, #T668), was used for staining. To visualize ΔΨ_m_ profile, naive ESCs were stained with 25 nM of TMRM directly on the tissue culture plate at 37 ^o^C, 5% CO_2_ for 15 min. Nuclei was stained with Hoechst 33342 (Thermo Fisher Scientific, #H3570, 1:2000). Live-cell imaging was performed using a Nikon X1 Yokogawa Spinning Disk Confocal (Nikon Instruments Inc.) (see below).

### Embryoid body (EB) differentiation

The EB medium consists of the KnockOut™ DMEM medium, 15% FBS, 2 mM Glutamine, 1X Non-essential amino acids, 0.055 mM 2-mercaptoethanol and Penicillin-Streptomycin (1:100). 2000 FACS-sorted ESCs with high and low ΔΨ_m_ were resuspended into 200 µl EB medium and plated onto each well of the Nunclon^TM^ Sphera^TM^ 96U plate (day 0). EBs were cultured in suspension at 37 ^o^C and 5% CO_2_. Half of medium were replaced on day 1, followed by full medium change every other day from day 2. EBs in suspension at day 3 and 6 were collected for RT-qPCR analysis. At day 7, EBs were transferred to 24-well tissue culture plates coated with Gelatin for additional 7 days. Differentiating cells were then fixed by 4% PFA/PBS for immunostaining to detect ectoderm (TUJ1), mesoderm (SMA) and endoderm (GATA6) markers as described below.

### Mathematical simulation modeling for mitochondrial activity

Flow cytometry data of TMRM staining for bulk naive ESCs were used for curve fitting. The distribution of TMRM fluorescence intensity exhibited multimodal features that are captured by a combination of n gaussian distributions using the R package mixtools^57^. The probability of high ΔΨ_m_ and low ΔΨ_m_ ESCs were obtained using the Gaussian model. To simulate the dynamics of mitochondrial activity through cell divisions, we constructed a mathematical model based on following assumptions: 1) the mitochondrial activity for the parental cells was generated from a normal distribution with the same mean and standard deviation from the bulk ESCs; 2) the range of mitochondrial activities for the progenies was calculated using the following formula, where *a* is the activity for each daughter: *a* = (max(1, a*runif(1,*m*,1)), *a*, a*runif(1,1,*n*)); 3) the activity for each daughter cell had three possible outcomes (I, J, K):

- I: the activity was decreased by multiplying a by a value between *m* and 1 and the lowest value was set to be 1; runif(1, *m*, 1) generated a value from a uniform distribution between *m* and 1;
- J: the activity remained at a;
- K: the activity was increased by multiplying a by a value between 1 and *n*; runif(1, 1, *n*) generated a value from a uniform distribution between 1 and *n*.

Using this model, the experimental data were applied to estimate the activity fluctuation coefficient, *t*. Most low ΔΨ_m_ daughter cells had increased (*Pk* = 0.8) activity and these cells regained the mitochondrial heterogeneity within 2–3 cell divisions. By comparing the difference in absolute value between the mean activity of daughter and parental cell populations, the activity fluctuation coefficient was estimated as 1.25, which means daughter cells regain similar parental ΔΨ_m_ distribution within 3 cell divisions if mitochondrial activity alters ~25% per division.

### Gene knockdown by siRNAs

To ensure the complete knockdown, we used the Dharmacon ON-TARGETplus siRNA SMARTPool that contains a mixture of four different siRNAs to target all transcript/splice variants of *Mat2a*. siRNA transfections were performed with the DharmaFECT 1 Transfection Reagent (Dharmacon) using reverse transfection protocol according to manufacturer’s instructions. Briefly, 2 × 10^4^ naive ESCs per 24-well culture area were transfected with either Dharmacon ON-TARGETplus Mouse *Mat2a* siRNA SMARTPool or ON-TARGETplus Non-targeting Control Pool at a final concentration of 40 nM. 18 h post transfection, medium change was performed, and cells were maintained for another 2 days prior to sample collection for subsequent analyses.

### Cell proliferation and cell cycle analysis

About 5000 FACS-sorted ESCs were replated onto Geltrex-coated 24-well tissue culture plates. Cells were dissociated into single cell by StemPro Accutase, stained with Trypan Blue 0.4% (Thermo Fisher Scientific, #15250061) and counted by hemocytometer every day for 3 consecutive days. For cell cycle analysis, cells were stained with 5 µg of Hoeschst 33342 (Thermo Fisher Scientific, #62249) and 1 µg of Pyronin Y (SIGMA ALDRICH, #213519) as previously described^5^. Stained cells were centrifuged, resuspended in ice-cold staining solution and subjected to flow cytometry analysis using the Propel Bigfoot Cell Sorter. Data were further analyzed by the FlowJo 10.8.1 (FlowJo LLC).

### *Elf5* promoter methylation analysis

Genomic DNA was extracted and 0.5 µg of genomic DNA per sample was subjected to bisulfite conversion using the Methylation-Gold^TM^ Kit (ZYMO RESEARCH, #D5005). About 4 µl cDNA was PCR amplified with primers^35^: Forward-TTTGTAGTTTGAGTATTTTGGTG; Reverse-ACCTTTCCACTCTAAACACCCAAA. Next-generation sequencing was performed at Center for Computational and Integrative Biology (CCIB) DNA Core at Massachusetts General Hospital. The raw paired-end bisulfite sequencing reads were processed using Trim Galore. The refined reads were aligned to the mouse genome (mm10) using Bismark^58^ with default settings. To determine the methylation levels of individual CpG sites within the regions of interest, the “bismark_methylation_extractor” function was employed to calculate the percentage of methylated CpG over the total calls for each specific site. A robust coverage exceeding 100× was attained for each examined CpG sites. The methylation levels were visualized using a violin plot in the ggplot2 package and a nonparametric Mann-Whitney test was employed for statistical analysis.

### Immunofluorescence

Cells were fixed with 4% paraformaldehyde (PFA, Ted Pella Inc. #18505)/PBS for 15 min at room temperature for histone antibodies or 30 min at 4 ^o^C for other antibodies. Cells were then washed three times with PBS, permeabilized in 0.25% Triton X-100/PBS (PBST) for 30 min at 4 ^o^C and blocked in blocking solution (10% Goat serum (Cell Signaling Technology, #5425) or 10% Donkey serum (SIGMA ALDRICH, #D9663), 0.1% BSA and 0.01% Tween20 in PBS) for 1 h at 4 ^o^C. Primary antibody incubation was performed at 4 ^o^C overnight. For detection of 5 mC, fixed cells were permeabilized with 0.4% TritonX-100/PBS for 30 min at 4 ^o^C, denatured with 2 N HCl for 30 min at room temperature, and neutralized with 100 mM *Tris*-HCl (pH 8.0) for 10 min at room temperature. After incubation with primary antibodies, cells were washed with 0.1% PBST three times prior to secondary antibody incubation at room temperature for 1 h, followed by 0.1% PBST wash for three times. Nuclei were stained with DAPI (Thermo Fisher Scientific, #62248, 1:1000) diluted in 0.1% PBST at room temperature for 10 min. Images were captured by the Leica Stellaris 5 Inverted confocal microscope (Leica Microsystems) with a 40× dry, 60× oil or 100× oil objectives across 0.5 µm stacks. Fluorescence was excited with a 405 nm UV laser (DAPI), a 488 nm laser (Alexa Fluor488) and a 561 nm laser (Alexa Fluor555). Images were also captured by the IX73 microscope system (Olympus). Image data were further processed by either Leica software or ImageJ software^59^.

### Antibodies for IF

The following primary antibodies were used: Mouse anti-OCT3/4 (Santa Cruz Biotechnology, Clone C-10, #sc-5279, 1:50), Rabbit anti-CDX2 (BioGenex, Clone EP25, #NU777-5UC, 1:100), Mouse anti-CDX2 (BioGenex, Clone CDX2-88, #MU392A-5UC, 1:100), Rabbit anti-EOMES (Abcam, #ab23345, 1:100), Mouse anti-SMA (SIGMA ALDRICH, Clone 1A4, #A2547, 1:200), Mouse anti-TUJ1 (Biolegend, Clone TUJ1, #801201, 1:200), Goat anti-GATA6 (R&D Systems, #AF1700, 1:100), Mouse anti-5mC (Millipore, Clone 33D3, #MABE146, 1:200), Rabbit anti-H3K27me3 (Millipore, #07-449, 1:100) and Rabbit anti-H3K9me3 (Abcam, #ab8898, 1:100). The secondary antibodies used were: Goat anti-Mouse IgG FITC-conjugated (Thermo Fisher Scientific, #62-6511, 1:50), Goat anti-Mouse IgG Alexa Fluor^R^ 555 (Abcam, #ab150114, 1:1000), Donkey anti-Goat IgG Alexa Fluor^TM^ 488 (Thermo Fisher Scientific, #A11055, 1:1000), Donkey anti-Mouse IgG Alexa Fluor^TM^ 555 (Thermo Fisher Scientific, #A31570, 1:1000), Donkey anti-Rabbit IgG Alexa Fluor^TM^ 555 (Thermo Fisher Scientific, #A31572, 1:1000), and Donkey anti-Rabbit IgG Alexa Fluor^TM^ 488 (Thermo Fisher Scientific, #A21206, 1:1000).

### Quantitative reverse transcription PCR (RT-qPCR) analysis

Total RNAs were extracted by the RNeasy mini kit. cDNA was synthesized using the SuperScriptTM III First-Strand Synthesis System with oligoT primer (Thermo Fisher Scientific, #18080051). qPCR was performed with the Radiant Green Lo-ROX qPCR Kit (Alkali Scientific Inc., #QS1020) on the 7500 Real Time PCR System (Applied Biosystems). The comparative cycling threshold (ΔΔ*Ct*) method was used to analyze the qPCR data. List of primers was shown in the Supplementary Data 8.

### Total RNA sequencing

Total RNAs were extracted by the RNeasy mini kit (QIAGEN). Genomic DNA was removed on-column by RNA-free DNase I for 15 min at room temperature. Total RNA amounts were determined by NANODROP 2000/2000c (Thermo Fisher Scientific) and assessed for quality using the TapeStation (Agilent, #5067-5576). Samples were prepared using the NEBNext Ultra II Directional RNA Library Prep Kit for Illumina (NEB, #E7760L), NEBNext rRNA Depletion Dit (Human/Mouse/Rat) (NEB, #E6310X), NEBNext Multiplex Oligos for Illumina (NEB, #E6440L) and ERCC RNA Spike-In control Mix (Thermo Fisher Scientific, #4456740). About 400 ng of total RNA was ribosomal depleted and fragmented and reverse transcribed. Samples underwent end repair and dA-Tailing step followed by ligation of NEBNext adapters. The libraries were pooled and sequenced on the Illumina NovaSeq 6000 with paired-end 150 bp at the University of Michigan core facility or AZENTA.

### Untargeted metabolomics analysis

The untargeted metabolomics analyses were performed as previously described^60^ with modifications. All samples were subjected to FACS sorting to isolate 5 × 10^5^ live cells. They were lysed using 1 ml of 80% cold methanol. Since the qESCs have lower biomass than cycling ESCs^5,6^, cell numbers were used to normalize metabolite fractions across samples before metabolite extraction. The extracts were incubated at −80 °C for 10 min and centrifugated at 14,000 rpm for 10 min at 4 °C. The supernatants were speed dried and re-suspended in 35 μl 50:50 MeOH:H2O mixture for LC-MS analysis. LC-MS analyses were performed using an Agilent Technologies Triple Quad 6470 LC-MS/MS system with a 1290 Infinity II LC Flexible Pump (Quaternary Pump), 1290 Infinity II Multisampler, 1290 Infinity II Multicolumn Thermostat with 6 port valve and 6470 triple quad mass spectrometers in both positive and negative acquisition mode as previously described^5,60^. The Agilent MassHunter Workstation Software was used for data acquisition. Agilent MassHunter Quantitative Analysis for QQQ (Version B.10.1.733.0) was used for initial raw data extraction. Results were then exported as CVS file. Metabolite counts were normalized by the total intensity of all metabolites to scale equal sample loading^5^. Finally, abundance of each metabolite in the sample was normalized divided by the median abundance levels across all samples for comparisons, statistical analyses and visualizations among metabolites. The statistical analysis was performed by the two-tailed Student’s *t test* with a significant threshold level of 0.05. Pathway enrichment analysis was performed using the MetaboAnalyst 5.0 (https://www.metaboanalyst.ca)^61^.

### Cleavage under targets and release using nuclease (CUT&RUN)

CUT&RUN analysis was performed with approximately 2 × 10^5^ naive ESCs for each library. Bead-bound cells were incubated overnight at 4 ^o^C with Rabbit anti-H3K4me3 (Millipore, #07-473) or Rabbit anti-H3K27me3 (Millipore, #07-449) antibody diluted in the antibody buffer (EDTA/Digitonin/wash buffer, EMD Millipore, #300410) (1 µg/500 µl). The beads were washed three times and incubated with 200 µl pA-MNase (0.7 ng/µl) for 1 h at 4 ^o^C. 2 mM of CaCl_2_ was added to activate MNase for 30 min on ice. The reaction was quenched by 2× stop buffer containing 340 mM NaCl, 20 mM EDTA, 4 mM EGTA, 0.02% Digitonin, 50 µg/ml RNase A (QIAGEN, #19101), 50 µg/ml glycogen (Roche, #10901393001) and 2 pg/ml Drosophila spike-in DNA at 37 ^o^C for 10 min. The soluble chromatins were converted to libraries using the NEBNext Ultra^TM^ II DNA library Prep kit (NEB, #E7645L). Libraries were subject to pair-ed sequencing on the Illumina NovaSeq 6000 instrument at the University of Michigan core facility.

### Assay for transposase-accessible chromatin with high-throughput sequencing (ATAC-seq)

Approximately 2 × 10^5^ naive ESCs were used for ATAC-seq analysis. Briefly, FACS-sorted cells were washed in cold D-PBS and resuspended in 50 µl of cold lysis buffer (10 mM *Tris*-HCl pH 7.4, 10 mM NaCl, 3 mM MgCl_2_, and 0.1% IGEPAL CA-630). Samples were spun at 500 *g* for 10 min, 4 ^o^C. Each cell pellet was resuspended in 50 µl of transposition reaction mix containing 25 µl of 2X TD buffer (Illumina, #FC-121-1030), 2.5 µl Tn5 transposase (Illumina, #FC-121-1030) and 22.5 µl nuclear free H_2_O. Tagmentation by Tn5 was performed at 37 ^o^C, 30 min. Libraries were sequenced on the Illumina NovaSeq 6000 instrument with paired end reads at the University of Michigan core facility.

### Bioinformatic analysis

*RNA-seq*. To perform data cleaning, the cutadapt (https://cutadapt.readthedocs.org/en/stable/) and FastQC (http://www.bioinformatics.babraham.ac.uk/projects/fastqc/) tools wrapped in Trim Galore (http://www.bioinformatics.babraham.ac.uk/projects/trim_galore/) were used to trim low quality bases (*Q* < 20) and adapter for raw sequences. RNA-seq data were mapped to the mm10 (GRCm38) genome of mouse and ERCC reference using Tophat2 (2.1.1)^62^. Duplicated reads for pair-end data were removed by SAMtools (v1.9)^63^. The bigwig files for visualization in Integrative Genomics Viewer (IGV)^64^ were generated from BAM files by using “bamCoverage” from deepTools3 (v3.2.1)^65^ with parameters “--outFileFormat bigwig --ignoreForNormalization chrM chrX chrY --skipNAs --bam input.accepted_hits.bam --outFileName output.bigwig --normalizeUsing RPKM --minMappingQuality 30 --binSize 20 --smoothLength 60 --ignoreDuplicates”.

Annotation of mRNA (GRCm38 v99) was downloaded from Ensembl database^66^. LncRNA annotation was downloaded from NONCODE database^67^ and repeat annotation was downloaded from Homer (v4.10)^68^. All three annotations were merged for gene annotation. Fragment (or template) count for each feature was performed by using featureCounts with parameters “-g ‘gene_id’ -p -M -O --fraction -C -F GTF -T 64 -s 0”^69^. Each reported alignment from a multi-mapping read (identified via “NH” tag) carried a fractional count of 1/*x*, instead of 1 (one), where *x* is the total number of alignments reported for the same read. Each overlapping feature received a fractional count of 1/*y*, where *y* is the total number of features overlapping with the read. Briefly, each alignment carried a fractional count of 1/(x*y). Fragment counts were transformed to fragments per kilobase of transcript per million (FPKM) by using edgeR^70^. Genes (or features) have more than three fragment counts in all samples were included. We employed RUVSeq^71^ to perform differential expression analysis by using ERCC as control genes with a likelihood ratio test from DESeq2^72^. Genes (or features) with the adjusted *P* value < 0.05 were reported. The following public datasets were used: GSM2711863 (ESC_2i)^41^, GSM2711865 (ESC_2i_EedKO)^41^, GSM838738 (MERVL-tdtomato^+^ and MERVL-tdtomato^-^)^19^, GSM1966767 (MERVL^+^_Zscan4^+^ and MERVL^+^_Zscan4^+^)^27^, GSM3384433 (CRISPRa_EV and CRISPRa_MERVL)^29^, GSM3110917 (NELFA^high^ and NELFA^low^)^30^, GSM1415501 (Zscan4^high^ and Zscan4^low^)^26^, GSM2279983 (Dux-GFP^+^, Dux-GFP^-^, Plus Dox-Dux, and Minus Dox-Dux)^28^, and GSE66582 (distinct developmental stages of preimplantation embryos)^25^.

To facilitate the comparisons between our and public RNA-seq data, we followed the “batch mean-centering” approach for batch effect removal^73^. Briefly, we have separately mean-centered the log_2_(FPKM + 1) value of each gene by subtracting the mean log_2_(FPKM + 1) across all samples. We used “1 - Spearman” correlation coefficient as the distance in the hierarchical clustering^7^. We computed the dissimilarity values with “as.dist” function in R, which were fed into hclust with “average” algorithm for agglomeration. The dendrogram were plotted by R.

#### CUT&RUN and ATAC-seq

Paired-end raw sequencing reads were trimmed with Trim Galore^74^ to remove adaptors and low-quality reads. Processed read (>20 bp) were mapped against mouse mm10 reference genome using bowtie2 (v2.4.2)^75^ with the following parameters “--local --very-sensitive-local --no-unal --no-mixed --no-discordant -q --phred33 -I 10 -X 700 --threads 12”. PCR duplicated reads were removed using samtools (v1.9)^63^. For H3K4me3, filtered BAM files for each sample were submitted to the callpeak function of MACS2 (v2.2.7.1)^76^ for peak regions calling with parameters “-p 1e-5 -g mm”. For H3K27me3 domains, peak calling was performed using SICER (v1.1)^77^ with default parameters for calling broad peaks. ATAC-seq paired-end raw sequencing reads were trimmed with Trim Galore^74^, then aligned to reference genome (mouse mm10) using Bowtie2 (v2.4.2)^75^ with parameters: “--local --very-sensitive-local --no-unal --no-mixed --no-discordant -q --phred33 -I 10 -X 2000 --threads 12”. The PCR duplicates from each dataset were removed using the rmdup function of samtools (v1.9)^63^. Peak calling was done using MACS2 (v2.2.7.1)^76^ with the parameters: “-B –nomodel –shif 100 –extsize 200”.

Differential binding analyses were performed using the edgeR package DiffBind (v3.4.11)^70,78^. Peaks derived from individual samples were loaded into dba.count function with minOverlap=1 and summits= FALSE.. The annotatePeak.pl script from Homer (v4.11.1)^68^ with default settings was employed for annotation. BAM files were merged for the biological replicates and converted to bigwig files for visualization in Integrative Genomics Viewer (IGV)^64^ by using the command “bamCoververage” of deeptools (v3.5.1)^79^ with parameters “-ignoreDuplicates -normalUsing RPKM -skipNonCoveredRegions -binSize 20”. Heatmaps were plotted using “computeMatrix” and “plotHeatmap” in deepTools (v3.5.1)^79^. Violin plots were performed by R package ggplot2.

De novo motifs were identified by the EPIGRAM^80^ pipeline. For H3K27me3, peaks with higher signals in high ΔΨ_m_ group were extracted as the target set and all the other H3K27me3 peaks were treated as the background set. Motifs were identified with following command line “perl epiplay-P.pl h3k27me3.target.bed h3k27me3.backgroup.bed 4 mm10 both 4G both”. TOMTOM (v5.4.1) (https://meme-suite.org/meme/tools/tomtom), an online motif comparison tool from the MEME suite^81^ was employed to identify the most matched motifs.

To determine the epigenome profiles near *MERVL*, deepTools computeMatrix was executed in the scale-region mode to calculate the signal density at each *MERVL* element using following parameters: “computeMatrix scale-regions -m 5000 -a 5000 -b 5000 -S bigwig.files -R bed.files”. For 2C genes, the signal density was also computed with computeMatrix in the scale-region mode in a normalized region 25 kb plus the 5 kb upstream to 5 kb downstream. DeepTools profiler was employed to generate the line plot to display the average values across the region. The violin plot was generated to show the intensity distribution by R package ggplot2, and a nonparametric Mann-Whitney test was used.

### Statistical analysis

Statistical analyses were performed by the two-tailed Student’s *t* test using GraphPad Prism 7.0 and 9.0 software. Data were presented as standard error of the mean (SEM). *P* values of less than 0.05 were considered statistically significant. GO term enrichment analysis for RNA-seq data was conducted using DAVID (https://david.ncifcrf.gov)^82^. GO terms with fold enrichment > 2 and the adjusted *P* value < 0.05 (Benjamini-Hochberg method) were considered as significant enrichment. GO analysis for CUT&RUN peaks was conducted using Panther Classification System Version 17.0 (http://pantherdb.org/). Statistical overrepresentation test was performed with “default settings”. Fisher’s Exact with false discovery rate (FDR < 0.05) multiple test correction was employed. GO terms (FDR < 0.05) for ATAC-seq used GREAT software with the Mouse Phenotype Single KO annotation^83^. Rules for the region-gene association included 5 kb upstream and 1 kb downstream of each gene.

### Reporting summary

Further information on research design is available in the Nature Portfolio Reporting Summary linked to this article.

### Supplementary information

Supplementary Information

Description of Additional Supplementary Files

Peer Review File

Supplementary Data 1

Supplementary Data 2

Supplementary Data 3

Supplementary Data 4

Supplementary Data 5

Supplementary Data 6

Supplementary Data 7

Supplementary Data 8

Reporting Summary

### Source data

Source Data

## Data Availability

The raw data from RNA-seq, ATAC-seq, CUT&RUN for H3K4me3, and H3K27me3, and bisulfite sequencing for the *Elf5* promoter have been deposited in the Gene Expression Omnibus (GEO) under the accession number GSE210915. The previous published data used in this study are available in the GEO under the accession numbers GSM2711863 (ESC_2i), GSM2711865 (ESC_2i_EedKO), GSM838738 (MERVL-tdtomato^+^ and MERVL-tdtomato^-^), GSM1966767 (MERVL^+^_Zscan4^+^ and MERVL^+^_Zscan4^+^), GSM3384433 (CRISPRa_EV and CRISPRa_MERVL), GSM3110917 (NELFA^high^ and NELFA^low^), GSM1415501 (Zscan4^high^ and Zscan4^low^), GSM2279983 (Dux-GFP^+^, Dux-GFP^-^, Plus Dox-Dux and Minus Dox-Dux), and GSE66582 (distinct developmental stages of preimplantation embryos). All source and processed data generated in this study, along with individual replicate values, are provided in the Supplemental Information and Source Data file. Source data are provided with this paper.

## Code availability

The source codes for mathematical simulation modeling are available at Zenodo [https://zenodo.org/records/10633128].
