# Author Correction: Quiescence enables unrestricted cell fate in naive embryonic stem cells

**DOI:** 10.1038/s41467-024-46566-4

**Published:** 2024-03-12

**Authors:** Le Tran Phuc Khoa, Wentao Yang, Mengrou Shan, Li Zhang, Fengbiao Mao, Bo Zhou, Qiang Li, Rebecca Malcore, Clair Harris, Lili Zhao, Rajesh C. Rao, Shigeki Iwase, Sundeep Kalantry, Stephanie L. Bielas, Costas A. Lyssiotis, Yali Dou

**Affiliations:** 1 Department of Medicine, Norris Comprehensive Cancer Center, University of Southern California, Los Angeles, CA 90033 USA; 2 Department of Molecular and Integrative Physiology, University of Michigan Medical School, Ann Arbor, MI 48109 USA; 3 Institute of Medical Innovation and Research, Peking University Third Hospital, Beijing, China; 4 Department of Human Genetics, University of Michigan Medical School, Ann Arbor, MI 48109 USA; 5 Department of Ophthalmology & Visual Sciences, W.K. Kellogg Eye Center, University of Michigan, 1000 Wall St., Ann Arbor, MI 48105 USA; 6 Beaumont Hospital, Wayne, 33155 Annapolis St., Wayne, MI 48184 USA; 7 Present Address: Department of Molecular and Integrative Physiology, University of Michigan Medical School, Ann Arbor, MI 48109 USA

**Keywords:** Pluripotency, Embryonic stem cells, Quiescence

Correction to: *Nature Communications* 10.1038/s41467-024-46121-1, published online 26 February 2024

In this article the author name Rajesh C. Rao was incorrectly written as Rajesh Rao. The original article has been corrected.

