# Supplementary material for: Quiescence enables unrestricted cell fate in naive embryonic stem cells
Source: Nat Commun. 2024 Feb 26;15:1721. doi: 10.1038/s41467-024-46121-1 (PMC10897426; doi:10.1038/s41467-024-46121-1)
Supplement: Supplementary file 1 — Supplementary Information [file 41467_2024_46121_MOESM1_ESM.pdf]

Supplementary Information

Quiescence Enables Unrestricted Cell Fate in Naive Embryonic Stem Cells

Le Tran Phuc Khoa[1,#], Wentao Yang[1], Mengrou Shan[2], Li Zhang[2], Fengbiao Mao[3], Bo Zhou[4], Qiang Li[5], Rebecca Malcore[4], Clair Harris[4], Lili Zhao[6], Rajesh Rao[5], Shigeki Iwase[4], Sundeep Kalantry[4], Stephanie L. Bielas[4], Costas A. Lyssiotis[2] and Yali Dou[1]*

[1]Department of Medicine, Norris Comprehensive Cancer Center, University of Southern California, Los Angeles, California 90033, USA

[2]Department of Molecular and Integrative Physiology, University of Michigan Medical School, Ann Arbor, Michigan 48109, USA

[3]Institute of Medical Innovation and Research, Peking University Third Hospital, Beijing, China

[4]Department of Human Genetics, University of Michigan Medical School, Ann Arbor, Michigan 48109, USA

[5]Department of Ophthalmology & Visual Sciences, W.K. Kellogg Eye Center, University of Michigan, 1000 Wall St., Ann Arbor, MI 48105, USA

[6]Beaumont Hospital, Wayne, 33155 Annapolis St, Wayne, MI 48184

*Correspondence: yalidou@usc.edu

[#]Present address: Department of Molecular and Integrative Physiology, University of Michigan Medical School, Ann Arbor, Michigan 48109, USA

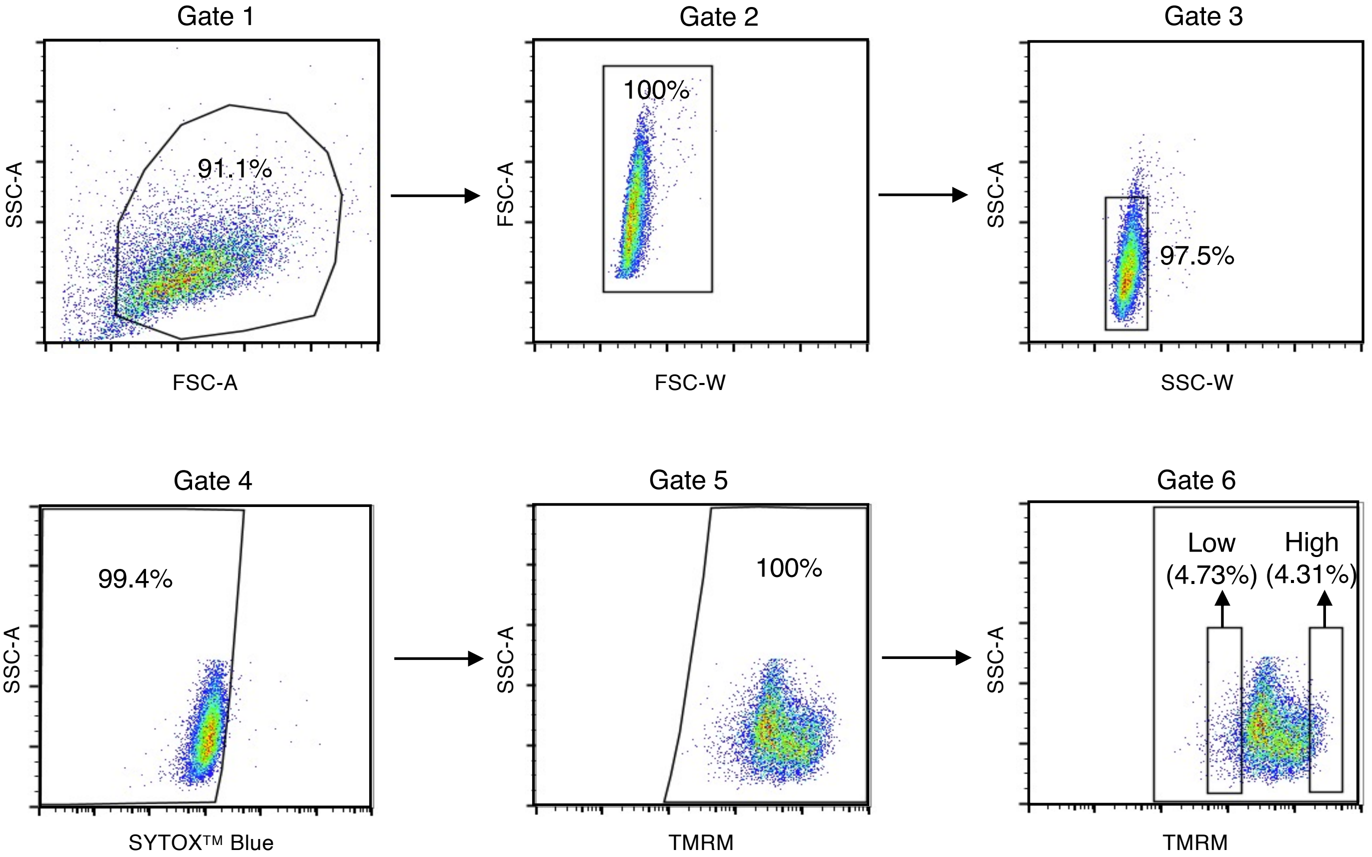

Supplementary Fig. 1: A representative gating strategy for flow cytometry. Cell population was defined by the SSC-A vs. FSC-A plot (Gate 1), followed by doublet removal by the FSC-A vs. FSC-W and SSC-A vs. SSC-W plots (Gate 2 and 3). Dead cells were then removed by SYTOX^{TM} Blue (Gate 4). SYTOX^{TM} Blue-negative cell population was used to gate for TMRM (Gate 5). Cell population with low and high TMRM signals were set for subsequent analyses (Gate 6).

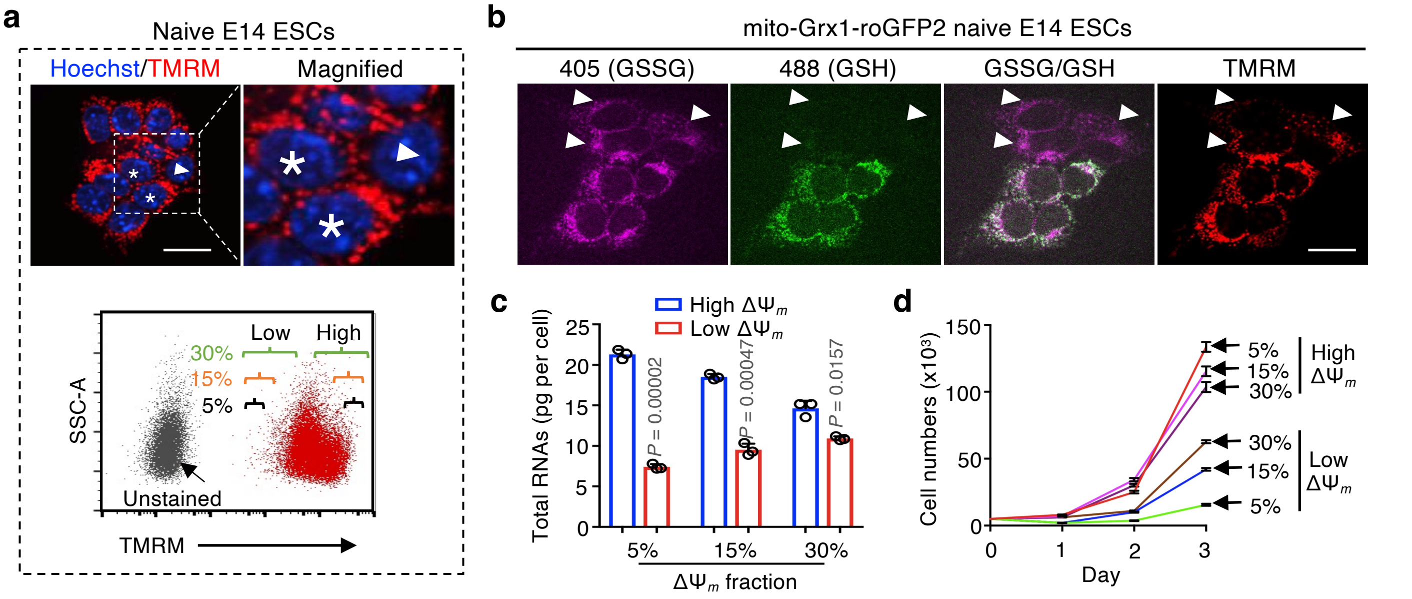

e

P values comparing proliferation rate (high vs. low ΔΨ$_m$)

ΔΨ$_m$	Day 1	Day 2	Day 3
5%	1.28×10^{-8}	1.53×10^{-9}	2.22×10^{-11}
15%	1.71×10^{-8}	7.97×10^{-9}	3.04×10^{-9}
30%	0.94	6.52×10^{-9}	1.45×10^{-6}

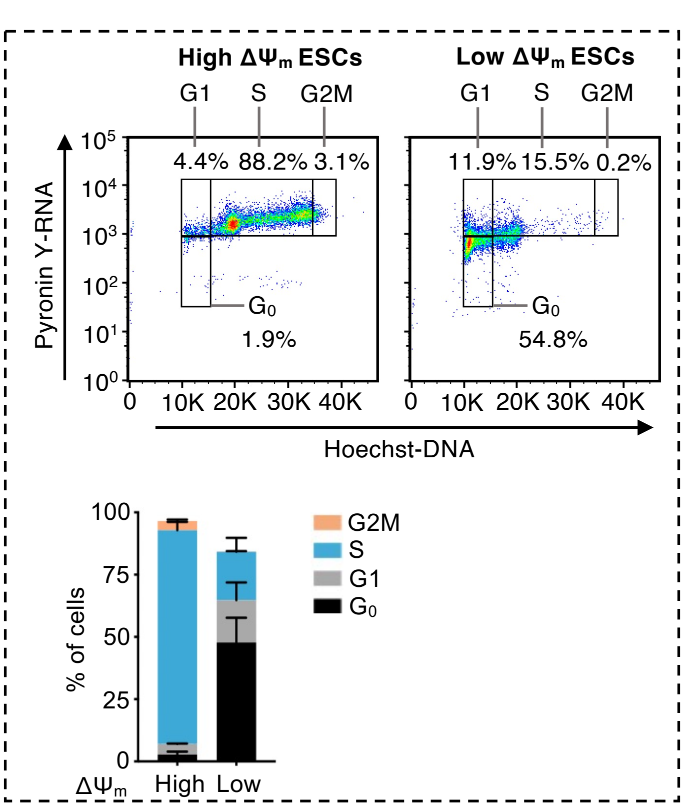

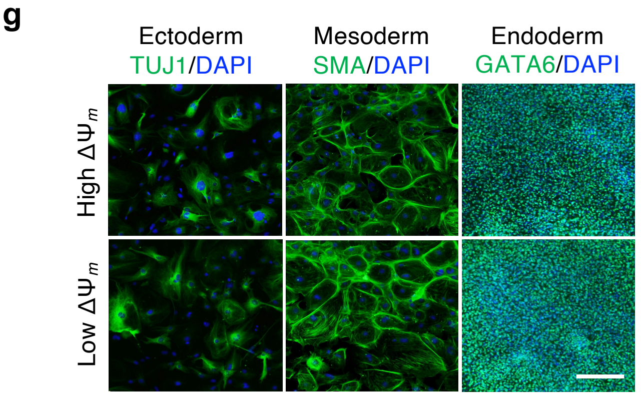

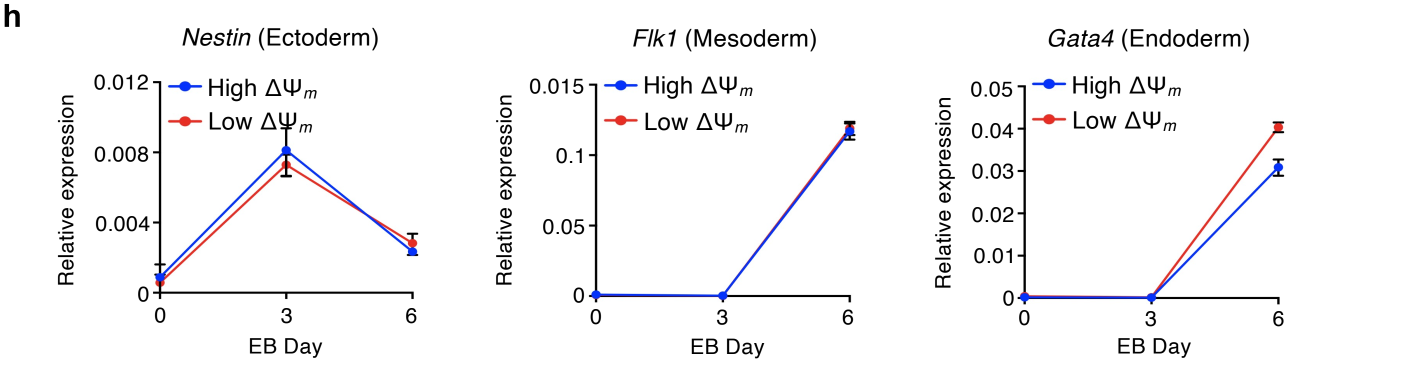

Supplementary Fig. 2: The spontaneous qESCs are pluripotent. a Top, live cell staining of naive ESCs with TMRM (red) and nuclei (Hoechst blue). Scale bar, 20 *μ*m. Asterisk and closed arrowhead indicate high and low TMRM (ΔΨ$_m$) cells, respectively. Bottom, FACS plot showing ΔΨ$_m$ profile and the strategy for sorting naive ESCs with distinct TMRM fluorescence intensity. Images and FACS plots were representatives from three independent experiments. **b** Live-cell staining of mito-Grx1-roGFP2 naive E14 ESCs with TMRM. Scale bar, 20 *μ*m. Closed arrowheads indicate cells that have an increase of GSSG/GSH ratio with concurrent decrease of TMRM signals. Images were representatives from three independent experiments. **c** Total RNAs/cell in naive ESCs with distinct

$\Delta\Psi_m$ fractions as denoted in **(a)**. Data were presented as mean ± SEM from three independent FACS sorting. *P* values were calculated using the two-sided Student's *t*-test. **d** Growth curves of naive ESCs with distinct $\Delta\Psi_m$ states. Data were presented as mean ± SEM from 6 biological replicates. See Supplementary Fig. 1e for statistical analysis. **e** *P* values of data shown in Supplementary Fig. 1d. The *P* values were determined by the two-sided Student's *t*-test. **f** Top, representative FACS plots showing cell cycle analysis in high and low $\Delta\Psi_m$ ESCs. Bottom, quantification of data shown from the top. Data were mean ± SEM from two independent FACS sorting samples. **g** Immunofluorescence of three-germ layer markers for embryoid body (EB) outgrowth derived from high and low $\Delta\Psi_m$ ESCs. Scale bar, 200 μm. Images were representatives from three independent experiments. **h** RT-qPCR analysis for representative genes as indicated on top. Relative mRNA expression was normalized against that of *H2A*. Data were presented as mean ± SEM from three technical replicates. Source data are provided as a Source Data file.

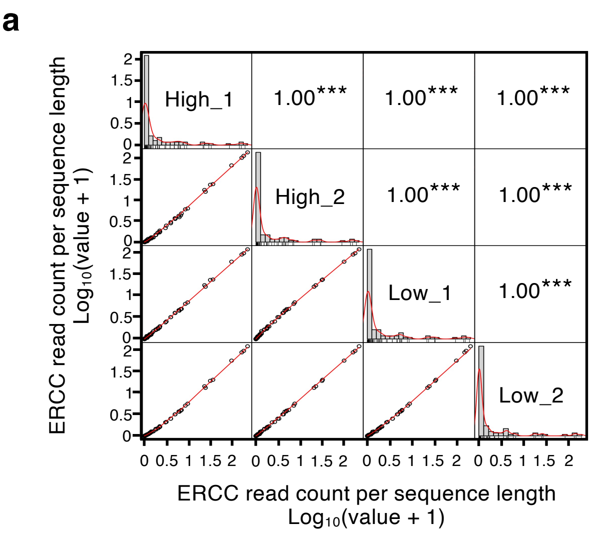

b

Sample		ERCC	Mouse	Ratio
High $\Delta\Psi_m$	High_1	783789	108236960	0.007241
	High_2	508392	93042143	0.005464
Low $\Delta\Psi_m$	Low_1	683364	112486956	0.006075
	Low_2	429030	76517484	0.005607

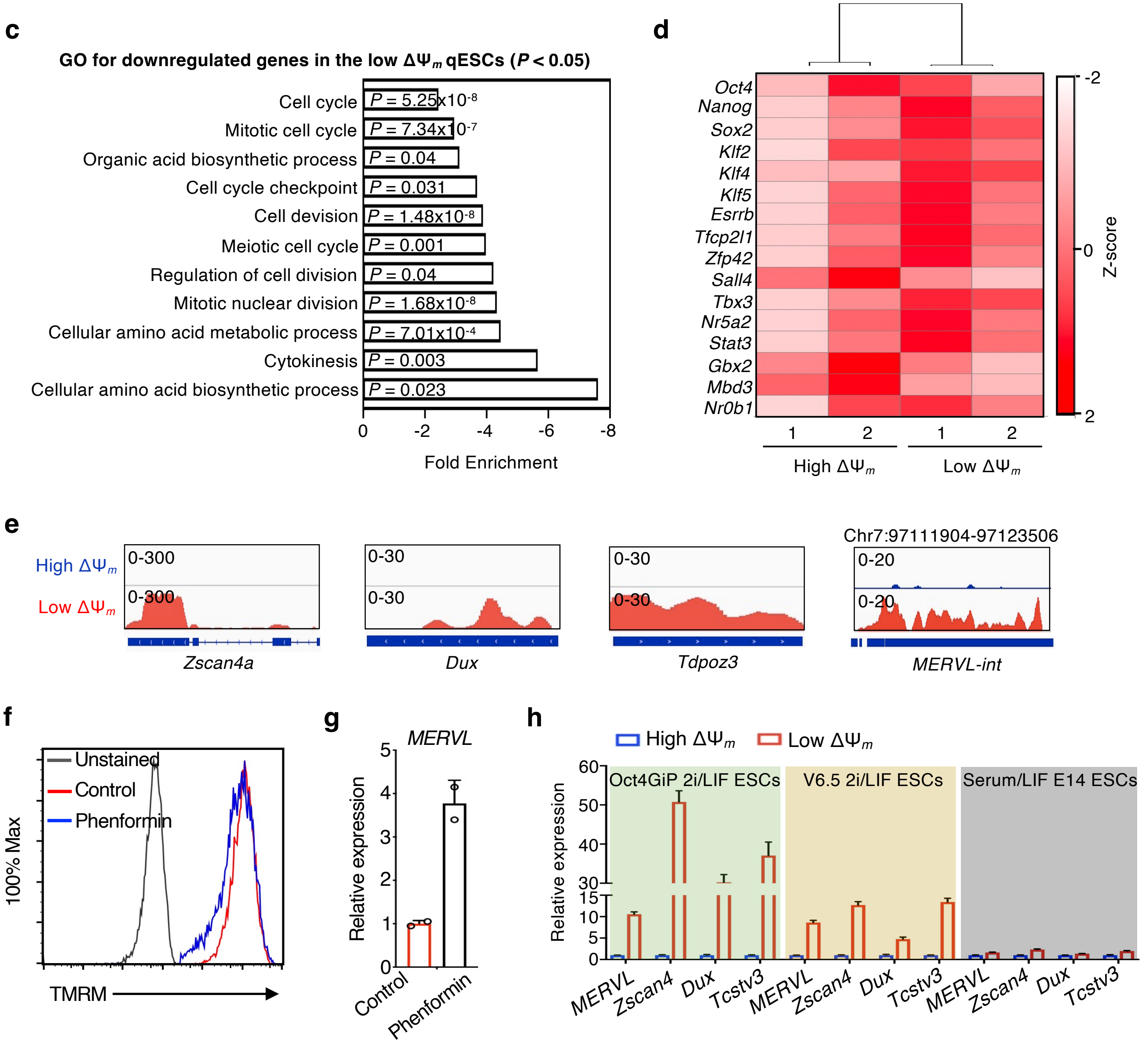

Supplementary Fig. 3: Transcriptome analysis for high and low $\Delta\Psi_m$ ESCs. a ERCC sequence abundance showing a highly linear trend across the samples. Diagonal entries in the figure matrix denote the density of read counts. **b** Ratio of ERCC/Mouse genome sequence reads across the samples. **c** Gene ontology (GO) analysis for genes significantly downregulated in the low $\Delta\Psi_m$ qESCs. GO terms with fold enrichment > 2 and the *P* value < 0.05 (defined by the Benjamini-Hochberg method) were considered as significant enrichment. **d** Heatmap showing unchanged expression levels of pluripotency genes. FPKM values were from RNA-seq data of biological duplicates. **e** Integrative Genomics Viewer (IGV) of RNA-seq for 2C genes and *MERVL* in high and low $\Delta\Psi_m$

ESCs. **f** A representative FACS plot from two biological replicates showing TMRM staining in naive ESCs treated with control or Phenformin. **g** RT-qPCR analysis for *MERVL* in control versus Phenformin-treated naive ESCs. Relative mRNA expression was normalized against that in the control naive ESCs, which was arbitrarily set 1. Data were presented as mean ± SEM from two biological replicates. **h** RT-qPCR analysis for *MERVL* and 2C genes in high and low $\Delta\Psi_m$ ESCs under 2i/LIF or Serum/LIF culture condition. Relative mRNA expression from three technical replicates was normalized against that in high $\Delta\Psi_m$ ESCs, which was arbitrarily set 1. Data were presented as mean ± SEM from one representative FACS sorting for each cell line. Source data are provided as a Source Data file.

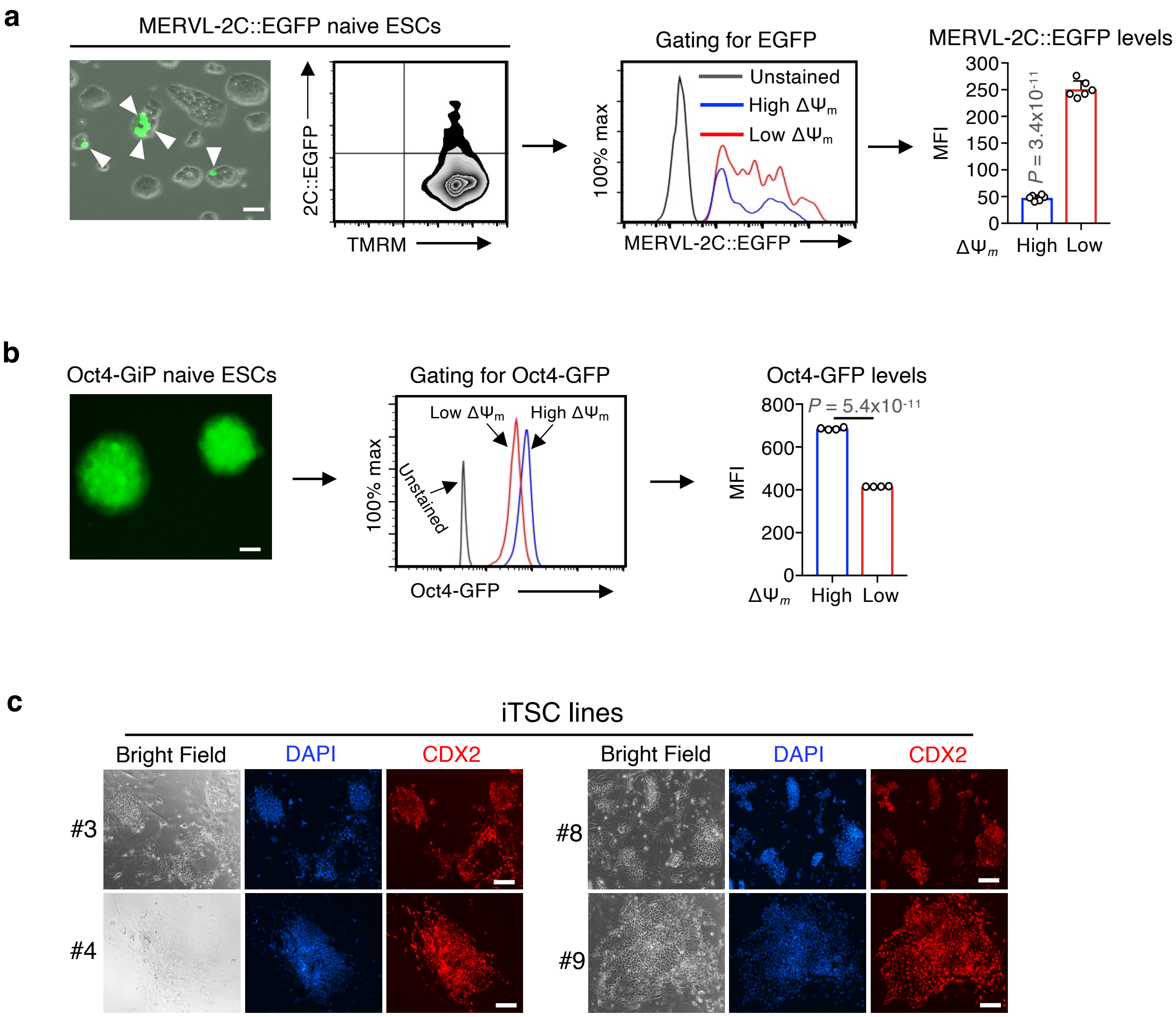

Supplementary Fig. 4: The spontaneous qESCs exhibit defined features of unrestricted cell fate. a Left, representative images of MERVL-2C::EGFP naive ESCs. Closed arrowheads denote GFP+ cells. Scale bar, 200 μm. Middle, FACS plots showing gating strategy for determining MERVL expression in naive ESCs with top or bottom 5% of TMRM fluorescence intensities. Right, quantified GFP signal intensities were mean ± SEM of 6 biological replicates. MFI, mean fluorescence intensity. **b** Left, representative images of Oct4-GiP naive ESCs. Scale bar, 200 μm. Middle, FACS plot showing OCT4 expression in naive ESCs with top or bottom 5% of TMRM fluorescence intensities. Right, quantified GFP signal intensities were mean ± SEM of 4 biological replicates. **c** Bright-field and immunofluorescence images of CDX2 (red) and Nuclei (DAPI blue) in the iTSC lines. Scale bar, 200 μm. Images were representatives from two independent experiments. The numbers on the left indicate the cell lines. *P* values for (**a)** and (**b)** were analyzed with the two-sided Student's *t*-test. Source data are provided as a Source Data file.

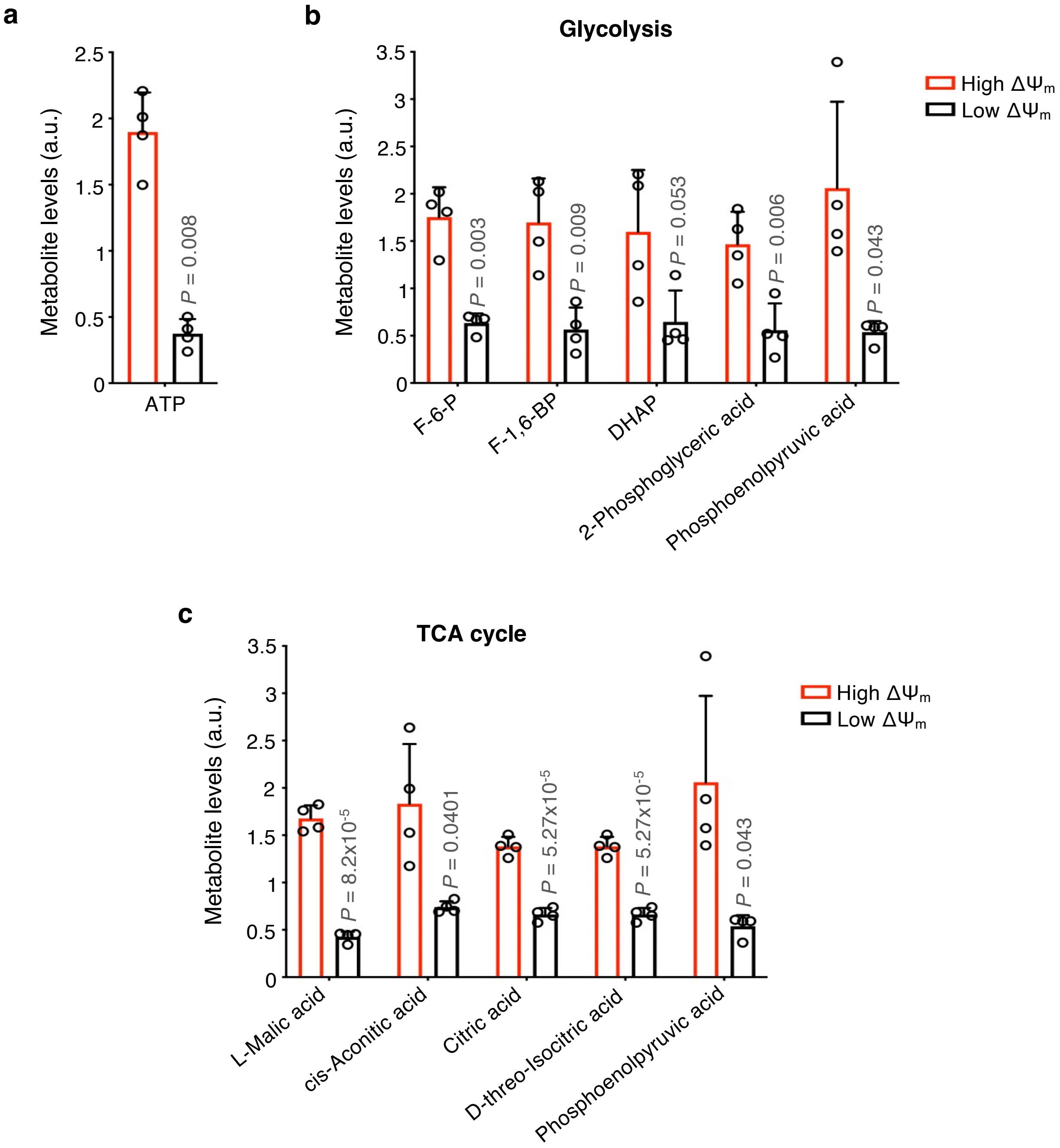

Supplementary Fig. 5: Metabolomics analysis in high and low $\Delta\Psi_m$ ESCs. a, b, c Levels of ATP (**a**) and metabolites associated with glycolysis (**b**) and TCA cycle (**c**) were presented as mean ± SEM from four biological replicates. a.u., arbitrary unit. *P* values were determined by the two-sided Student's *t*-test. F-6-P, Fructose-6-phosphate; F-1,6-BP, Fructose-1,6-biphosphate; DHAP, Dihydroxyacetone phosphate. Source data are provided as a Source Data file.

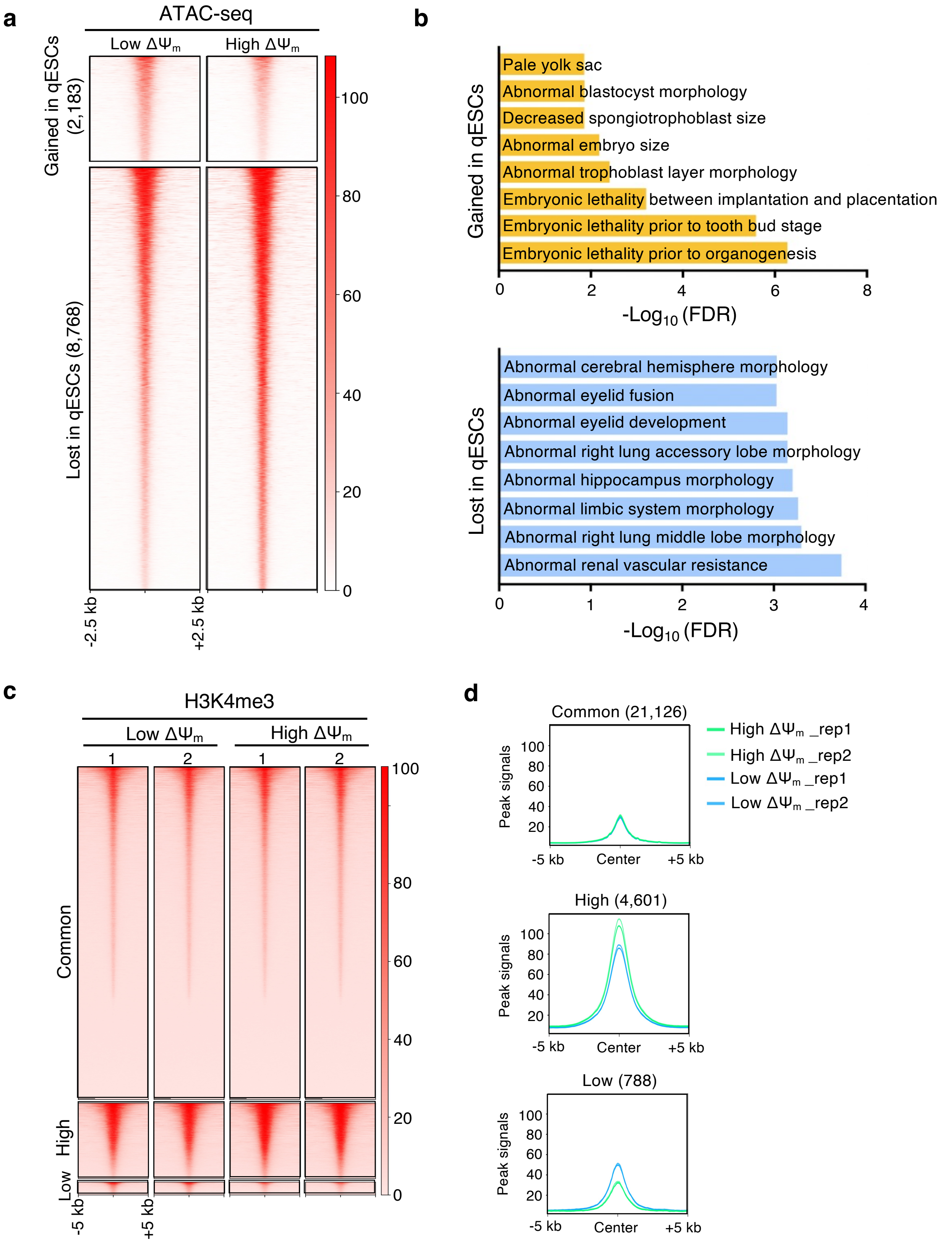

Supplementary Fig. 6: Epigenetic analysis of high and low $\Delta\Psi_m$ ESCs. **a** Heatmaps of ATAC-seq signals in high and low $\Delta\Psi_m$ ESCs from biological duplicates. Peaks were categorized as those gained or lost in the low $\Delta\Psi_m$ qESCs relative to high $\Delta\Psi_m$ ESCs. The number of peaks called in each category was shown on left. **b** GO term analysis for peaks called in **(a)**. GO analysis was performed using GREAT software with Mouse Phenotype Single KO annotation. GO terms with [FDR] < 0.05 were selected. **c** Heatmaps of H3K4me3 CUT&RUN signals in high and low $\Delta\Psi_m$ ESCs from biological duplicates. Peaks were categorized as common peaks (equal signals in high and low $\Delta\Psi_m$ ESCs), high or low-specific peaks (higher signals in high or low $\Delta\Psi_m$ ESCs). **d** Line plots showing averaged peak signals for peak categories in **(c)**. The numbers above each plot represent the number of peaks called in each category.

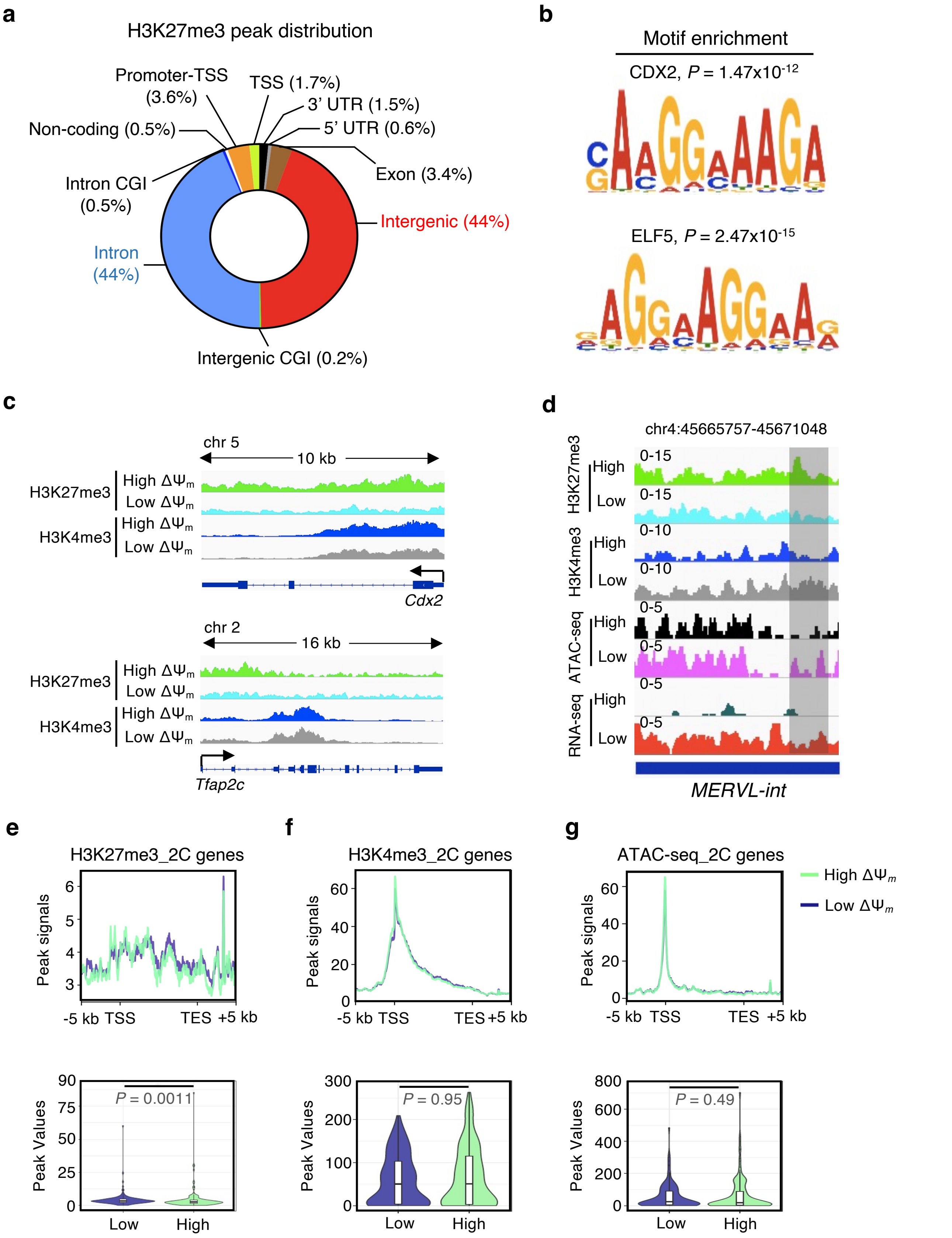

Supplementary Fig. 7: CUT&RUN analysis for H3K27me3 in high and low $\Delta\Psi_m$ ESCs. a Pie charts illustrating genomic distribution of 10,076 peaks called in Fig. 5a. **b** Motif analysis for chromatin regions with H3K27me3 peak reduction in the low $\Delta\Psi_m$ qESCs. **c** IGV of H3K27me3 and H3K4me3 CUT&RUN at indicated genes in high and low $\Delta\Psi_m$ ESCs. Decrease of H3K27me3 signals at the trophoblast lineage gene loci (*Cdx2* and *Tfap2c*) were detected. The signals of H3K4me3 at the same loci remained relatively unchanged. **d** IGV of H3K27me3, H3K4me3, ATAC-seq and RNA-seq at a representative *MERVL* locus. **e**, **f**, **g** Top, line plots showing slight decrease of H3K27me3 but unaltered levels of H3K4me3 and ATAC-seq signals at 145 2C genes in the low $\Delta\Psi_m$ ESCs relative to high $\Delta\Psi_m$ ESCs. The 2C gene list used in this analysis was similar to that shown in Fig. 1b. Bottom, violin box plots representing the peak values of the top plots, which show the median, 1st, 3rd quartiles, lower adjacent value, upper adjacent value, and outside points. *P* values were determined by a non-parametric Mann-Whitney test.

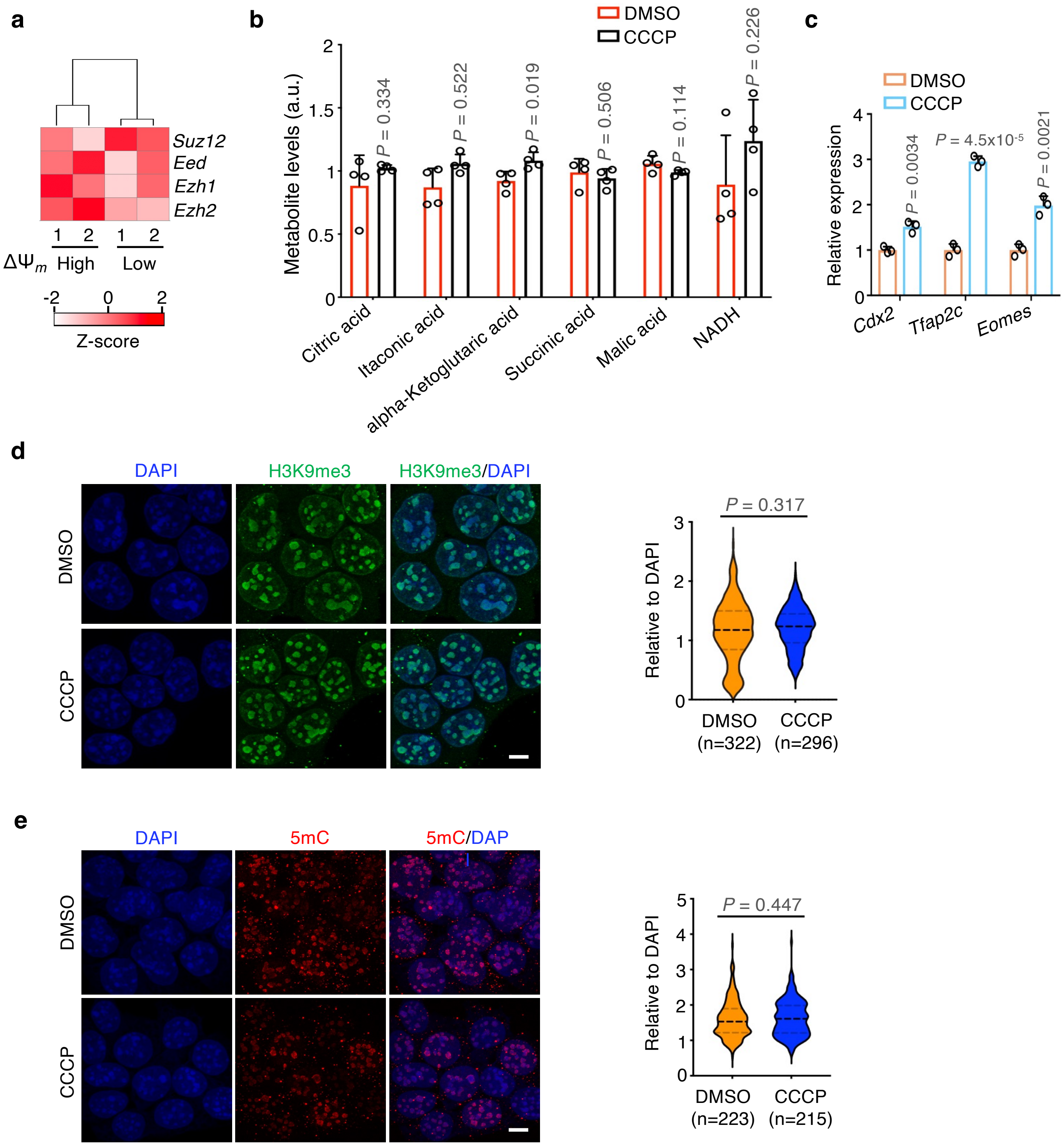

Supplementary Fig. 8: Multi-omics analysis in CCCP-treated naive ESCs. **a** Heatmap (Z-score) showing expression levels of the indicated PRC2 core subunits in high and low $\Delta\Psi_m$ ESCs. RNA-seq data from biological duplicates were shown. **b** Levels of TCA cycle metabolites in naive ESCs treated with DMSO or CCCP. Data were presented as mean ± SEM from four biological replicates. a.u., arbitrary unit. *P* values were determined by the two-sided Student's *t*-test. **c** RT-qPCR analysis for trophoblast genes in naive ESCs treated with DMSO or CCCP. Data were mean ± SEM from three biological replicates. *P* values were calculated using the two-sided Student's *t*-test. **d** Left, immunofluorescence of H3K9me3 (green) and nuclei (DAPI blue) in naive ESCs treated with DMSO or CCCP. Scale bar, 10 μm. Right, H3K9me3 fluorescence signals at foci were quantified relative to DAPI from three biological replicates. n, number of foci. Violin plots show the median, 1st and 3rd quartiles. **e** Left, immunostaining of 5mC (red) and nuclei (DAPI blue) in naive ESCs treated with DMSO or CCCP. Scale bar, 10 μm. Right, 5mC fluorescence signals at foci were quantified relative to DAPI from three biological replicates. n, number of foci. Violin plots show the median, 1st and 3rd quartiles. *P* values for (**d**) and (**e**) were calculated by a non-parametric Mann-Whitney test. Source data are provided as a Source Data file.

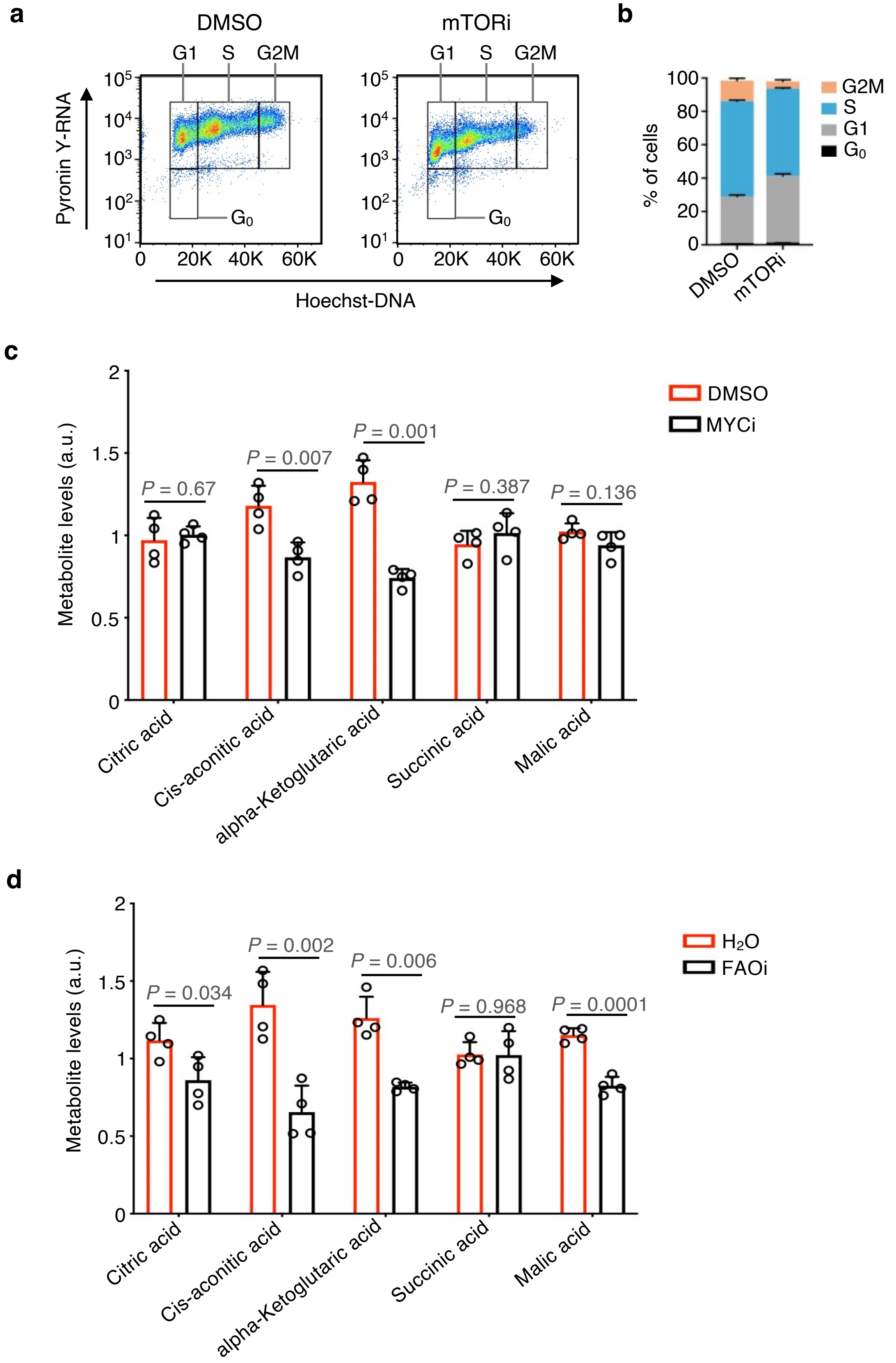

Supplementary Fig. 9: Effects of mTORi, MYCi or FAOi on cell cycle and metabolomics profile of naive ESCs. a Representative FACS plots showing cell cycle analysis in naive ESCs treated with DMSO or mTOR inhibitor. **b** Quantification of **(a)**. Data were mean ± SEM from three technical replicates. **c, d** Levels of TCA cycle metabolites in MYCi **(c)** and FAOi **(d)** relative to DMSO and H_2O, respectively. Data were presented as mean ± SEM from four biological replicates. a.u., arbitrary unit. *P* values were determined by the two-sided Student's *t*-test. Source data are provided as a Source Data file.

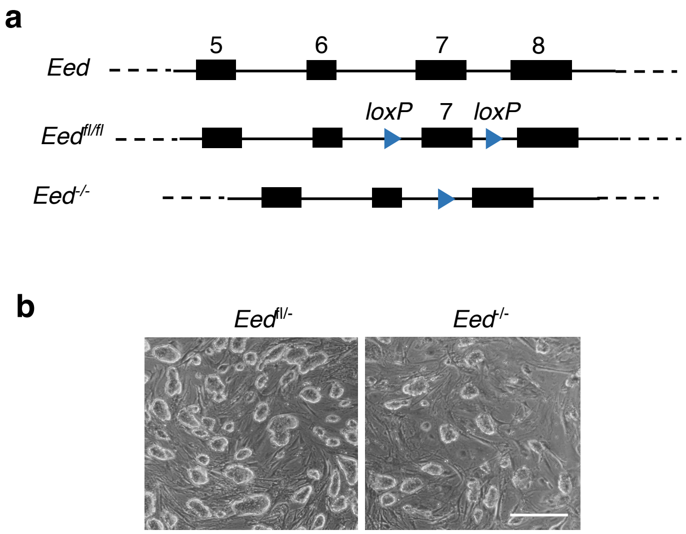

Supplementary Fig. 10: Generation of *Eed*$^{-/-}$ naive ESCs. **a** Diagram representation of wild-type (*Eed*), targeted (*Eed*$^{fl/fl}$) and null alleles (*Eed*$^{-/-}$). Numbers above the black bars indicate exon number. **b** Representative images of bright field for *Eed*$^{fl/-}$ and *Eed*$^{-/-}$ naive ESCs were from two independent experiments. One representative cell line for each genotype was shown. Scale bar, 200 μm.

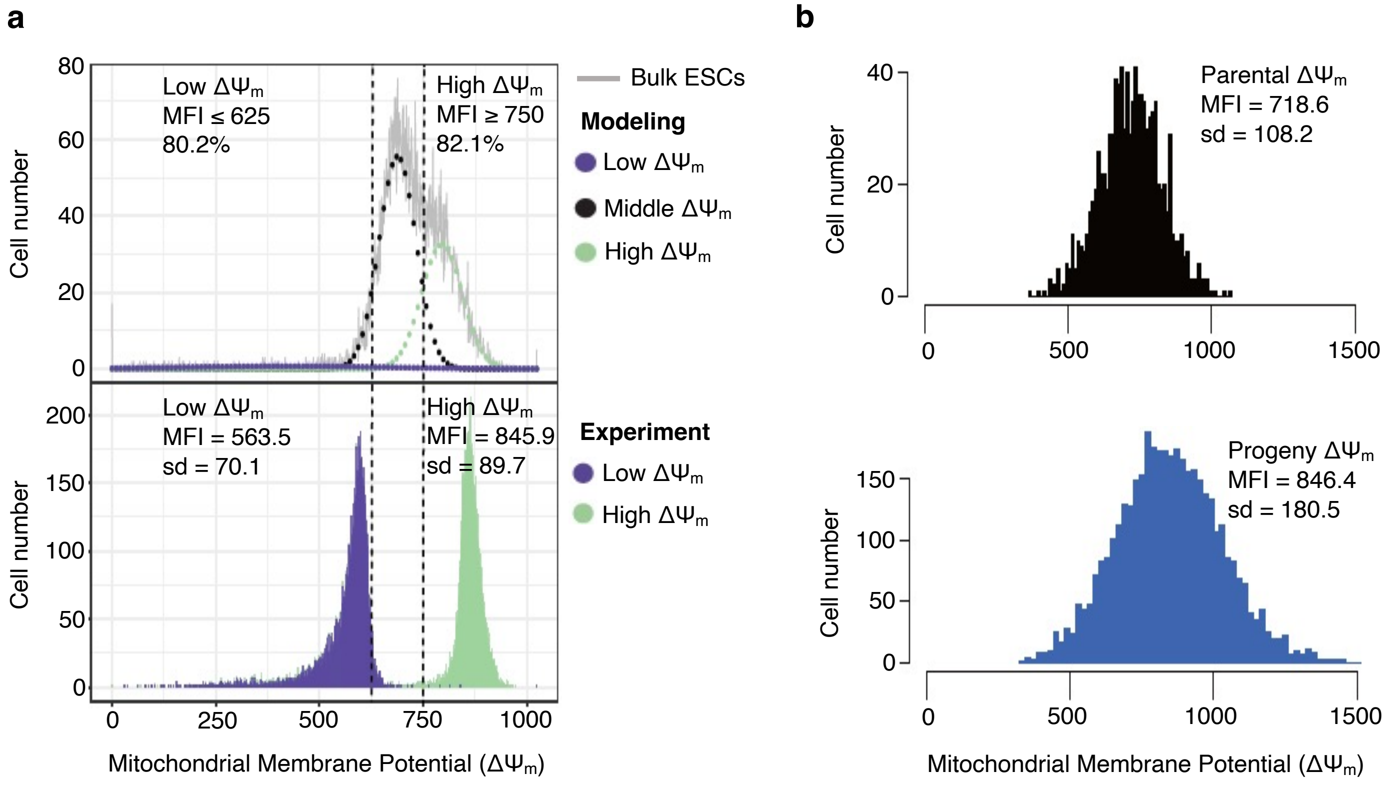

Supplementary Fig. 11: Mathematical simulation modeling for mitochondrial activity. a Top, distribution of mitochondrial activity based on TMRM live-cell staining ($\Delta\Psi_m$) for bulk naive ESCs (gray line). The dash-dotted lines represent three components based on the gaussian mixture model: high $\Delta\Psi_m$ (green dots), middle $\Delta\Psi_m$ (black dots), and low $\Delta\Psi_m$ (blue dots). Bottom, distribution of mitochondrial activity for low $\Delta\Psi_m$ (blue histogram on the left), and high $\Delta\Psi_m$ (green histogram on the right) was obtained by the experimental FACS sorting. The black dashed lines show $\Delta\Psi_m$ threshold in the bulk naive ESCs. **b** Simulation of stochastic mitochondrial activity distribution for parental (top) and progeny (bottom) of isolated low $\Delta\Psi_m$ ESCs after two cell divisions. For all panels, X-axis denotes $\Delta\Psi_m$ levels, whereas Y-axis represents cell number. MFI, mean fluorescence intensity; sd, standard deviation. Source data are provided as a Source Data file.
