# Supplementary material for: Quiescence enables unrestricted cell fate in naive embryonic stem cells
Source: Nat Commun. 2024 Feb 26;15:1721. doi: 10.1038/s41467-024-46121-1 (PMC10897426; doi:10.1038/s41467-024-46121-1)
Supplement: Supplementary file 2 — Description of Additional Supplementary Files [file 41467_2024_46121_MOESM2_ESM.pdf]

Description of Additional Supplementary Files

File Name: Supplementary Data 1
Description: Transcriptomic analysis in high and low ΔΨm ESCs.

File Name: Supplementary Data 2
Description: Metabolomics analysis in high and low ΔΨm ESCs.

File Name: Supplementary Data 3
Description: ATAC-seq analysis in high and low ΔΨm ESCs.

File Name: Supplementary Data 4
Description: CUT&RUN analysis for H3K27me3 in high and low ΔΨm ESCs.

File Name: Supplementary Data 5
Description: CUT&RUN analysis for H3K4me3 in high and low ΔΨm ESCs.

File Name: Supplementary Data 6
Description: Metabolomics analysis in naive ESCs treated with FAOi, MYCi or CCCP.

File Name: Supplementary Data 7
Description: Transcriptomic analysis in siMat2a vs. siControl and Eed-/- vs. Eedfl/- naive ESCs.

File Name: Supplementary Data 8
Description: Primer sequences for RT-qPCR.
