# Supplementary material for: Quiescence enables unrestricted cell fate in naive embryonic stem cells
Source: Nat Commun. 2024 Feb 26;15:1721. doi: 10.1038/s41467-024-46121-1 (PMC10897426; doi:10.1038/s41467-024-46121-1)
Supplement: Supplementary file 12 — Reporting Summary [file 41467_2024_46121_MOESM12_ESM.pdf]

nature portfolio

Corresponding author(s): Yali Dou

Last updated by author(s): January 31, 2024

Reporting Summary

Nature Portfolio wishes to improve the reproducibility of the work that we publish. This form provides structure for consistency and transparency in reporting. For further information on Nature Portfolio policies, see our Editorial Policies and the Editorial Policy Checklist.

Statistics

For all statistical analyses, confirm that the following items are present in the figure legend, table legend, main text, or Methods section.

n/a	Confirmed	
☐	☒	The exact sample size (n) for each experimental group/condition, given as a discrete number and unit of measurement
☐	☒	A statement on whether measurements were taken from distinct samples or whether the same sample was measured repeatedly
☐	☒	The statistical test(s) used AND whether they are one- or two-sided<br>*Only common tests should be described solely by name; describe more complex techniques in the Methods section.*
☒	☐	A description of all covariates tested
☒	☐	A description of any assumptions or corrections, such as tests of normality and adjustment for multiple comparisons
☐	☒	A full description of the statistical parameters including central tendency (e.g. means) or other basic estimates (e.g. regression coefficient) AND variation (e.g. standard deviation) or associated estimates of uncertainty (e.g. confidence intervals)
☐	☒	For null hypothesis testing, the test statistic (e.g. F, t, r) with confidence intervals, effect sizes, degrees of freedom and P value noted<br>*Give P values as exact values whenever suitable.*
☒	☐	For Bayesian analysis, information on the choice of priors and Markov chain Monte Carlo settings
☒	☐	For hierarchical and complex designs, identification of the appropriate level for tests and full reporting of outcomes
☒	☐	Estimates of effect sizes (e.g. Cohen's d, Pearson's r), indicating how they were calculated

Our web collection on statistics for biologists contains articles on many of the points above.

Software and code

Policy information about availability of computer code

Data collection	Flow cytometry data were collected by the Sony SH800 Cell Sorter or the Propel Bigfoot Cell Sorter. Live-cell imaging data was collected using a Nikon X1 Yokogawa Spinning Disk Confocal. Immunofluorescence images were captured by the Leica Stellaris 5 Inverted confocal microscope or the IX73 microscope system. Metabolite data was collected by an Agilent Technologies Triple Quad 6470 LC-MS/MS system with a 1290 Infinity II LC Flexible Pump (Quaternary Pump), 1290 Infinity II Multisampler, 1290 Infinity II Multicolumn Thermostat with 6 port valve and 6470 triple quad mass spectrometer. RNA-seq, ATAC-seq, and CUT&RUN data were collected by the Illumina NovaSeq 6000 instrument with paired-end reads.

Data analysis	Flow cytometry data was analyzed by the FlowJo v10.8.1.<br><br>For metabolite data, the Agilent MassHunter Workstation Software LC/MS Data Acquisition for 6400 Series Triple Quadrupole MS with Version B.08.02 was used for compound optimization and sample data acquisition. Agilent MassHunter Quantitative Analysis for QQQ with Version B.10.1.733.0 was used for initial raw data extraction. Results were then exported as CVS file for further analysis. Integration of peak areas of detected compounds was performed by software analysis and further validated by manual inspection. The statistical analysis was performed by the two-tailed Student's t-test with a significant threshold level of 0.05 using the GraphPad Prism v7.0 or v9.0. Pathway enrichment analysis was performed using the MetaboAnalyst 5.0.<br><br>RNA-seq, ATAC-seq, and CUT&RUN data were analyzed by the following softwares: TrimGalore (v0.6.4), Tophat2 (v2.1.1), SAMtools (v1.9), deepTools3 (v3.2.1), deeptools (v3.5.1), Homer (v4.10), Homer (v4.11.1), RUVSeq, DESeq2, bowtie2 (v2.4.2), samtools (v1.9), MACS2 (v2.2.7.1), SICER (v1.1), DiffBind (v3.4.11), EPIGRAM, TOMTOM (v5.4.1).<br><br>The raw paired-end bisulfite sequencing reads were processed using Trim Galore, Bismark13, the ggplot2 package.

For manuscripts utilizing custom algorithms or software that are central to the research but not yet described in published literature, software must be made available to editors and reviewers. We strongly encourage code deposition in a community repository (e.g. GitHub). See the Nature Portfolio guidelines for submitting code & software for further information.

Data

Policy information about availability of data

All manuscripts must include a data availability statement. This statement should provide the following information, where applicable:

- Accession codes, unique identifiers, or web links for publicly available datasets
- A description of any restrictions on data availability
- For clinical datasets or third party data, please ensure that the statement adheres to our policy

The raw RNA sequencing, ATAC sequencing, CUT&RUN sequencing for H3K4me3, and H3K27me3, and bisulfite sequencing for Elf5 promoter have been deposited in the Gene Expression Omnibus (GEO) under the accession number GSE210915. The previously published sequencing data analyzed here are available in the GEO under the accession numbers GSM2711863 (ESC_2i), GSM2711865 (ESC_2i_EedKO), GSM838738 (MERVL-tdtomato+ and MERVL-tdtomato-), GSM1966767 (MERVL+_Zscan4+ and MERVL+_Zscan4+), GSM3384433 (CRISPRa_EV and CRISPRa_MERVL), GSM3110917 (NELFAhigh and NELFAlow), GSM1415501 (Zscan4high and Zscan4low), GSM2279983 (Dux-GFP+, Dux-GFP-, Plus Dox-Dux and Minus Dox-Dux), and GSE66582 (distinct developmental stages of preimplantation embryos).
All processed data generated in this study, along with individual replicate values, are provided in the Supplemental Information and Source Data file. Source data are provided with this paper.

Human research participants

Policy information about studies involving human research participants and Sex and Gender in Research.

Reporting on sex and gender	N/A
Population characteristics	N/A
Recruitment	N/A
Ethics oversight	N/A

Note that full information on the approval of the study protocol must also be provided in the manuscript.

Field-specific reporting

Please select the one below that is the best fit for your research. If you are not sure, read the appropriate sections before making your selection.

☒ Life sciences ☐ Behavioural & social sciences ☐ Ecological, evolutionary & environmental sciences

For a reference copy of the document with all sections, see nature.com/documents/nr-reporting-summary-flat.pdf

Life sciences study design

All studies must disclose on these points even when the disclosure is negative.

Sample size	No statistical methods were employed to predetermine the sample sizes. For all sequencing datasets, two independent biological replicates were used to determine P and FDR values. All cell biological experiments were performed in at least three biological replicates unless otherwise specified.

Data exclusions	No data exclusions
Replication	All attempts at replications were successful. All experiments were repeated at least twice, but usually many more. Details on replications were clearly specified in the figure legends.
Randomization	Samples were allocated into groups based on treatments and genotypes. No further randomization was required.
Blinding	Since data were collected and analyzed using objective quantitative methods, blinding is not relevant to this study.

Reporting for specific materials, systems and methods

We require information from authors about some types of materials, experimental systems and methods used in many studies. Here, indicate whether each material, system or method listed is relevant to your study. If you are not sure if a list item applies to your research, read the appropriate section before selecting a response.

Materials & experimental systems

n/a	Involved in the study	
☐	☒	Antibodies
☐	☒	Eukaryotic cell lines
☒	☐	Palaeontology and archaeology
☐	☒	Animals and other organisms
☒	☐	Clinical data
☒	☐	Dual use research of concern

Methods

n/a	Involved in the study	
☐	☒	ChIP-seq
☐	☒	Flow cytometry
☒	☐	MRI-based neuroimaging

Antibodies

Antibodies used

The following primary antibodies were used: Mouse anti-OCT3/4 (Santa Cruz Biotechnology, Clone C-10, #sc-5279, 1:50), Rabbit anti-CDX2 (BioGenex, Clone EP25, #NU777-5UC, 1:100), Mouse anti-CDX2 (BioGenex, Clone CDX2-88, #MU392A-5UC, 1:100), Rabbit anti-EOMES (Abcam, #ab23345, 1:100), Mouse anti-SMA (SIGMA ALDRICH, Clone 1A4, #A2547, 1:200), Mouse anti-TUJ1 (Biolegend, Clone TUJ1, #801201, 1:200), Goat anti-GATA6 (R&D Systems, #AF1700, 1:100), Mouse anti-5mC (Millipore, Clone 33D3, #MABE146, 1:200), Rabbit anti-H3K27me3 (Millipore, #07-449, 1:100) and Rabbit anti-H3K9me3 (Abcam, #ab8898, 1:100). All antibodies were used for Immunofluorescence, except the H3K4me3 antibody was used for CUT&RUN analysis.

The secondary antibodies used were: Goat anti-Mouse IgG FITC-conjugated (Thermo Fisher Scientific, #62-6511, 1:50), Goat anti-Mouse IgG Alexa FluorR 555 (Abcam, #ab150114, 1:1000), Donkey anti-Goat IgG Alexa FluorTM 488 (Thermo Fisher Scientific, #A11055, 1:1000), Donkey anti-Mouse IgG Alexa FluorTM 555 (Thermo Fisher Scientific, #A31570, 1:1000), Donkey anti-Rabbit IgG Alexa FluorTM 555 (Thermo Fisher Scientific, #A31572, 1:1000), and Donkey anti-Rabbit IgG Alexa FluorTM 488 (Thermo Fisher Scientific, #A21206, 1:1000).

Validation

All antibodies used in this study are commercially available. They has been validated by the manufacturer as following websites:
Mouse anti-OCT3/4 https://www.scbt.com/p/oct-3-4-antibody-c-10?gclid=Cj0KCQiA1NebBhDDARIsAANiDD39PoSqWdfG8BE4Wwa7NG4THrPe3JHvndcGrTKMcdp1g7U2hKYOnqkaAtBKEALw_wcB
Rabbit anti-EOMES https://www.abcam.com/en-it/products/primary-antibodies/anti-tbr2-eomes-antibody-ab23345
Mouse anti-CDX2 https://biogenex.com/product/anti-cdx-2/?v=7516fd43adaa
Mouse anti-SMA https://www.sigmaaldrich.com/US/en/product/sigma/a2547
Mouse anti-TUJ1 https://www.biolegend.com/en-us/explore-new-products/purified-anti-tubulin-beta-3-tubb3-antibody-11580
Goat anti-GATA6 https://www.rndsystems.com/products/human-gata-6-antibody_af1700
Mouse anti-5mC https://www.emdmillipore.com/US/en/product/Anti-5-methylcytosine-Antibody-clone-33D3,MM_NF-MABE146#
Rabbit anti-H3K27me3 https://www.emdmillipore.com/US/en/product/Anti-trimethyl-Histone-H3-Lys27-Antibody,MM_NF-07-449
Rabbit anti-H3K4me3 https://www.emdmillipore.com/US/en/product/Anti-trimethyl-Histone-H3-Lys4-Antibody,MM_NF-07-473
Rabbit anti-H3K9me3 https://www.abcam.com/histone-h3-tri-methyl-k9-antibody-chip-grade-ab8898.html
Goat anti-Mouse IgG FITC-conjugated https://www.thermofisher.com/antibody/product/Goat-anti-Mouse-IgG-H-L-Secondary-Antibody-Polyclonal/62-6511
Goat anti-Mouse IgG Alexa FluorR 555 https://www.abcam.com/goat-mouse-igg-hl-alexa-fluor-555-ab150114.html
Donkey anti-Goat IgG Alexa FluorTM 488 https://www.thermofisher.com/antibody/product/Donkey-anti-Goat-IgG-H-L-Cross-Adsorbed-Secondary-Antibody-Polyclonal/A-11055
Donkey anti-Mouse IgG Alexa FluorTM 555 https://www.thermofisher.com/antibody/product/Donkey-anti-Mouse-IgG-H-L-Highly-Cross-Adsorbed-Secondary-Antibody-Polyclonal/A-31570
Donkey anti-Rabbit IgG Alexa FluorTM 555 https://www.thermofisher.com/antibody/product/Rabbit-IgG-H-L-Highly-Cross-Adsorbed-Secondary-Antibody-Polyclonal/A-31572
Donkey anti-Rabbit IgG Alexa FluorTM 488 https://www.thermofisher.com/antibody/product/Donkey-anti-Rabbit-IgG-H-L-Highly-Cross-Adsorbed-Secondary-Antibody-Polyclonal/A-21206

Eukaryotic cell lines

Policy information about cell lines and Sex and Gender in Research

Cell line source(s)	Mouse embryonic stem cell (ESCs) lines used in this study include: male E14tg2a (E14) (ATCC, #CRL-1821TM), Oct4-GiP ESCs (a gift from Dr. Austin Smith, University of Exeter, United Kingdom), male V6.5 ESCs (Novus Biologicals, #NBP1-41162) and iCdx2 Elf5-2A-mCherry reporter ESCs (A gift from Dr. Janet Rossant, SickKids Research Institute, Canada).<br><br>Heterozygous (Eedfl/-) and null (Eed-/-) naive male ESC lines were derived from the 129/S1 background embryos.
Authentication	Commercial E14 and V6.5 ESC lines were authenticated by sequencing at ATCC and Novus Biologicals, respectively. Other cell lines were authenticated by gene expression analysis.
Mycoplasma contamination	All cell lines were routinely tested negative for mycoplasma
Commonly misidentified lines (See ICLAC register)	No misidentified cell lines were used in this study

Animals and other research organisms

Policy information about studies involving animals; ARRIVE guidelines recommended for reporting animal research, and Sex and Gender in Research

Laboratory animals	3-8-week-old female and 2-6-month-old male mice were used for all experiments. Mouse strain JF1 carries a conditional mutation of Eed gene in which its exon 7 was flanked by loxP sequences. These mice were generated and maintained onto the 129/S1 background.
Wild animals	No wild animals were used in this study.
Reporting on sex	Males and female mice used for generating E3.5 blastocysts were between 8 and 12 weeks of age.
Field-collected samples	Mice were maintained on 12 h light/dark cycle in the Transgenic Animal Model Core, University of Michigan. All animal procedures were reviewed and approved by the University of Michigan Institutional Animal Care and Use Committee.
Ethics oversight	All mice were handled according to the protocols approved by the University Committee on Use and Care of Animals (UCUCA) at the University of Michigan (protocol #PRO00004007 and PRO00006455).

Note that full information on the approval of the study protocol must also be provided in the manuscript.

ChIP-seq

Data deposition

☐ Confirm that both raw and final processed data have been deposited in a public database such as GEO.

☒ Confirm that you have deposited or provided access to graph files (e.g. BED files) for the called peaks.

Data access links<br>*May remain private before publication.*	https://www.ncbi.nlm.nih.gov/geo/query/acc.cgi?acc=GSE210915
Files in database submission	GSM6442914 TMRM_H3K27me3_high_1<br>GSM6442915 TMRM_H3K27me3_high_2<br>GSM6442916 TMRM_H3K4me3_High_1<br>GSM6442917 TMRM_H3K4me3_High_2<br>GSM6442918 TMRM_H3K27me3_Low_1<br>GSM6442919 TMRM_H3K27me3_Low_2<br>GSM6442920 TMRM_H3K4me3_Low_1<br>GSM6442921 TMRM_H3K4me3_Low_2
Genome browser session (e.g. UCSC)	No public available genome browser session

Methodology

Replicates	All CUT&RUN experiments for H3K4me3 and H3K27me3 were performed in two biological replicates.
Sequencing depth	Paired-ends, 25M sequencing reads for each sample
Antibodies	Rabbit anti-H3K27me3 (Millipore, #07-449), Rabbit anti-H3K4me3 (Millipore, #07-473)
Peak calling parameters	For H3K4me3, filtered BAM files for each sample were submitted to the callpeak function of MACS2 (v2.2.7.1)43 for peak regions

calling with parameters “-p 1e-5 -g mm”.

For H3K27me3 domains, peak calling was performed using SICER (v1.1)44 with default parameters for calling broad peaks.

Data quality	Paired-end raw sequencing reads were trimmed with Trim Galore40 to remove adaptors and low-quality reads before mapping to the mouse mm10 reference genome.
Software	Trim Galore, bowtie2 (v2.4.2), samtools (v1.9), MACS2 (v2.2.7.1), SICER (v1.1), DiffBind (v3.4.11), Homer (v4.11.1), deepTools (v3.5.1), TOMTOM (v5.4.1).

Flow Cytometry

Plots

Confirm that:

- [x] The axis labels state the marker and fluorochrome used (e.g. CD4-FITC).
- [x] The axis scales are clearly visible. Include numbers along axes only for bottom left plot of group (a 'group' is an analysis of identical markers).
- [x] All plots are contour plots with outliers or pseudocolor plots.
- [x] A numerical value for number of cells or percentage (with statistics) is provided.

Methodology

Sample preparation	ESCs were dissociated by StemPro Accutase at 37oC, 3 min and collected in pre-warmed FACS buffer (1X Phenol red-free HBSS, 10 mM HEPES and 2% FBS). Cells were then washed once with FACS buffer. Next, cells were resuspended into staining solution containing FACS buffer plus 25 nM Tetramethylrhodamine Methyl Ester (TMRM) and incubated at 37oC, 5% CO2 for 15 min. After staining, cells were washed once and resuspended in the culture medium containing 10 mM HEPES and SYTOXTM Blue for 10 min on ice. For cell cycle analysis, dissociated ESCs were stained with 5 µg of Hoeschst 33342 and 1 µg of Pyronin Y. Stained cells were centrifuged, resuspended in ice-cold staining solution, filtered through 40 µm sterile cell strainer and kept on ice until flow cytometry analysis.
Instrument	Sony SH800 Cell Sorter and the Propel Bigfoot Cell Sorter
Software	FlowJo 10.8.1 (FlowJo LLC).
Cell population abundance	After FACS sorting, sorted cells were re-checked for the purity of over 95%.
Gating strategy	To remove debris from subsequent analysis, cell population (over 80%) was defined by the SSC-A/FSC-A plot. Then, the FSC-A/FSC-W and SSC-A/SSC-W plots were used to remove doublets. Dead cells were removed by SYTOXTM Blue channel. Non-staining samples were always used to set the gate for all experimental conditions. Negative population were set at over 99.9%.

- [x] Tick this box to confirm that a figure exemplifying the gating strategy is provided in the Supplementary Information.
